# A FtsZ inhibitor-acinetobactin conjugate with enhanced cellular uptake in *Acinetobacter baumannii* acts synergistically in combination with PBP3-targeting antibiotics

**DOI:** 10.1371/journal.pone.0334409

**Published:** 2025-10-14

**Authors:** Eric J. Bryan, Zoltan Szekely, Yuxuan Wang, Huizhou Fan, Lucile Moynié, Jacques Y. Roberge, Daniel S. Pilch

**Affiliations:** 1 Department of Pharmacology, Rutgers Robert Wood Johnson Medical School, Piscataway, New Jersey, United States of America; 2 Department of Molecular Design and Synthesis, Office for Research, Rutgers University Core Services, Piscataway, New Jersey, United States of America; 3 Rosalind Franklin Institute, Harwell Science and Innovation Campus, Oxford, United Kingdom; East Carolina University Brody School of Medicine, UNITED STATES OF AMERICA

## Abstract

Multidrug resistance in the Gram-negative bacterial pathogen *Acinetobacter baumannii* is a global public health threat, highlighting a critical need for drug development. FtsZ is an essential cell division protein that is an appealing target for new antibacterial agents. Benzamide-based FtsZ inhibitors have been developed that exhibit potent activity against Gram-positive pathogens, but poor activity against *A. baumannii* and other clinically significant Gram-negative pathogens. In this connection, we have initiated a program to enhance activity of benzamide FtsZ inhibitors via conjugation to iron-coordinating siderophore moieties that promote cellular uptake through endogenous bacterial siderophore-iron uptake pathways. Here, we describe a second-generation FtsZ inhibitor-siderophore conjugate (RUP7), in which an oxazole-benzamide FtsZ inhibitor is conjugated to acinetobactin, a principal native siderophore of *A. baumannii*. In iron-limiting conditions that are typically present at common sites of *A. baumannii* infection, RUP7 exhibits significantly enhanced activity against a library of *A. baumannii* clinical isolates relative to the non-conjugated FtsZ inhibitor (RUP2) or a first-generation conjugate (RUP4) in which RUP2 has been conjugated to a chlorocatechol siderophore functionality. This enhanced activity is correlated with markedly improved cellular uptake, perhaps via the BauABCDEF acinetobactin-iron uptake system, the expression of which is highly upregulated in iron-limiting conditions. RUP7 exhibits significant bactericidal synergy against *A. baumannii* when combined with clinical antibiotics that target penicillin binding protein 3 (PBP3 or FtsI), including aztreonam, piperacillin:tazobactam, cefsulodin, and ceftazidime. In the aggregate, our results highlight the combination of design-optimized FtsZ inhibitor-siderophore conjugates and PBP3-targeting antibiotics as an appealing therapeutic strategy for the treatment of *A. baumannii* infections.

## Introduction

Multidrug resistance in clinically important bacterial pathogens is a worsening global public health problem. In 2019, bacterial antimicrobial resistance was associated with 4.95 million deaths globally and, without intervention, is predicted to nearly double to 8.22 million annually by 2050 [1–4]. The World Health Organization (WHO) has identified *Acinetobacter baumannii* as a Gram-negative bacterial pathogen of particular concern and being a critical priority for therapeutic drug development [5]. Globally, deaths related to resistant *A. baumannii* infections totaled over 400,000 in 2019, with resistance to standard-of-care clinical antibiotics being widespread [3]. These alarming trends highlight an urgent need to develop new and effective therapeutics to treat *A. baumannii* infections.

FtsZ is a highly conserved essential protein that functions as the primary coordinator for bacterial cell division [6,7]. FtsZ polymerizes into a structure known as the Z-ring at the midcell of a dividing bacterium, where it acts as a scaffold and temporal regulator for the recruitment of other division-associated proteins (collectively forming the divisome) [7,8]. Importantly, FtsZ is a highly druggable target, and multiple FtsZ inhibitors have been developed with potent activity against Gram-positive pathogens like methicillin-resistant *Staphylococcus aureus* (MRSA) [9–23]. By contrast, few FtsZ inhibitors have been associated with activity against *A. baumannii*, with the benzothiadiazole compound (C109) being one such inhibitor recently reported [24]. Among the FtsZ inhibitors reported to date, benzamide-based inhibitors have shown significant preclinical and clinical success against MRSA [9,10,12,13,15–23,25–27]. However, their activity against most Gram-negative isolates, including *A. baumannii*, is typically poor, due in large part to the deleterious impacts of diminished cellular influx as well as enhanced cellular efflux [14,23,26,28–31].

The outer membrane of Gram-negative bacteria is a significant barrier to the cellular uptake of benzamide-based FtsZ inhibitors as well as many other classes of antibiotics [28–30,32]. Siderophore conjugation to antibiotics offers an appealing approach for enhancing drug uptake into bacterial cells [33–35]. Siderophores are molecules synthesized and secreted by bacterial pathogens under limiting ferric iron (Fe^3+^) conditions [36–38]. Fe^3+^ is an essential metabolic cofactor for bacterial pathogens that is restricted in infection environments by the innate immune system of the host [36,37,39–41]. Siderophores directly coordinate Fe^3+^, with the resulting siderophore-Fe^3+^ complexes being internalized by the bacteria via dedicated uptake pathways [36–38].

**Fig 1:**
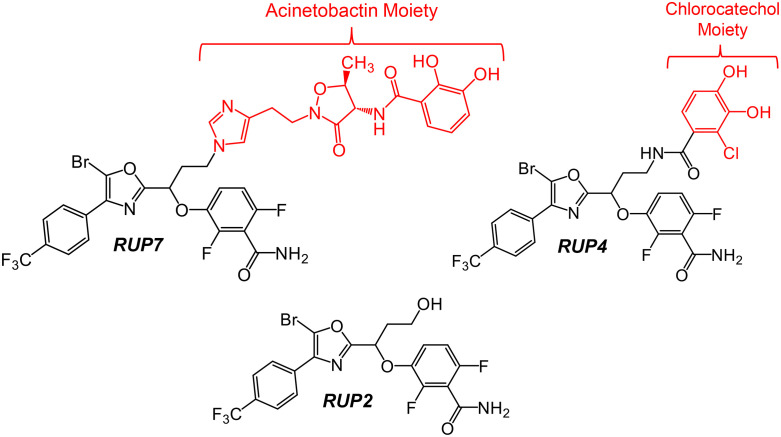
Chemical structures of RUP7, RUP4, and RUP2. The siderophore moieties are shown in red, while the FtsZ inhibitor functionalities are depicted in black.

We have initiated a program geared towards enhancing the cellular uptake and corresponding antibacterial activity of benzamide-based FtsZ inhibitors against Gram-negative pathogens through siderophore conjugation. Our previous structural studies on the interactions of oxazole-benzamide FtsZ inhibitors similar to RUP2 (whose structure is shown in Fig 1) with *S. aureus* FtsZ revealed the linker connecting the oxazole and difluorobenzamide rings to be a suitable site for tethering bulky substituents while still maintaining the capability to target FtsZ [42,43]. Using this structure-guided approach, we designed a BODIPY fluorophore conjugate to generate a fluorescent probe (termed BOFP) for the visualization of FtsZ in a broad range of both Gram-positive and Gram-negative bacterial pathogens [42]. We also developed a first-generation siderophore conjugate (RUP4) with a chlorocatechol substituent as the siderophore functionality tethered to RUP2 (see structure of RUP4 in Fig 1). RUP4 was associated with enhanced antibacterial activity against *Klebsiella pneumoniae*, with this activity being linked to the ability of the compound to coordinate Fe^3+^ and utilize innate siderophore uptake transporters to facilitate influx into *K. pneumoniae* cells [27]. However, RUP4 was associated with only modest antibacterial activity against *A. baumannii* [27].

Acinetobactin and fimsbactin are major native siderophores in *A. baumannii* [44,45]. Conjugation of fimsbactin analogs to antibiotics (e.g., daptomycin and β-lactam agents) whose targets are localized to the membrane or periplasm has been shown to enhance activity against *A. baumannii* [46–48]. By contrast, conjugation to antibiotics (e.g., ciprofloxacin) whose targets are cytoplasmic showed no enhancement of activity against *A. baumannii* [48]. Consistent with these observations, the Wencewicz and Skaar groups have suggested that acinetobactin is transported from the extracellular space into the cytoplasm via the BauABCDEF uptake transporter system, while fimsbactin appears to be transported only to the periplasm via the FbsN outer membrane uptake transporter [44,49], where it competes with acinetobactin for binding to the BauB periplasmic siderophore chaperone protein [49]. As FtsZ is primarily a cytoplasmic target, we hypothesized that conjugating acinetobactin to RUP2 instead of the chlorocatechol functionality in RUP4 would further enhance activity against *A. baumannii*. In this connection, through an elegant series of structure-activity relationship studies, Kim and coworkers identified the imidazole ring of acinetobactin as an appealing site for antibiotic conjugation [50]. To this end, we designed the second-generation FtsZ inhibitor-siderophore conjugate RUP7 (shown in Fig 1), in which RUP2 is tethered to the imidazole ring of acinetobactin. Here, we show that RUP7 exhibits enhanced antibacterial activity against a library of *A. baumannii* clinical isolates relative to both RUP4 and non-conjugated RUP2. Significantly, the enhanced activity of RUP7 is closely correlated to improved cellular uptake into *A. baumannii* cells, with this enhanced uptake likely being via a highly upregulated BauABCDEF transport system. We also show that RUP7 is associated with significant bactericidal synergy when combined with antibiotics that target penicillin-binding protein 3 (PBP3 or FtsI). Viewed as a whole, our results highlight the use of design-optimized FtsZ inhibitor-siderophore conjugates in combination with PBP3-targeting antibiotics as an appealing strategy for treating *A. baumannii* infections.

## Materials and methods

### Compound synthesis

RUP2 and RUP4 were synthesized as described previously [27]. RUP7 was synthesized as detailed in the Supporting Information (S1 Appendix).

### Bacterial strains and reagents

The following strains were obtained from the American Type Culture Collections (ATCC): *A. baumannii* 19606 and 17978, *K. pneumoniae* 13883, *Pseudomonas aeruginosa* 27853, *Escherichia coli* 25922, *Listeria monocytogenes* 19115, *Bacillus subtilis* 23857, and *Staphylococcus saprophyticus* 15305. *A. baumannii* clinical isolates (NR-13374, NR-13376, NR-13377, NR-13378, NR-13379, NR-13380, NR-13382, and NR-13385) and the *Staphylococcus aureus* (MRSA) clinical isolate (NR-46234) were obtained from BEI Resources. Mutant strains of *S. aureus* NR46234 expressing either G196S or N263K mutant FtsZ were generated as described previously [22]. Unless otherwise indicated, *A. baumannii* strains were grown in modified M9 media containing 6.8 g/L Na_2_HPO_4_, 3 g/L KH_2_PO_4_, 0.5 g/L NaCl, 1 g/L NH_4_Cl, 2 mM MgSO_4_, 0.1 mM CaCl_2_, 0.4% glucose, 0.2% low-iron casein amino acids, 16.5 µg/mL thiamine hydrochloride, and 0.2 µM FeCl_3_. Low-iron casein amino acids, tryptic soy agar (TSA), cation-adjusted Mueller Hinton (CAMH) media, brain-heart infusion (BHI) media, and agar were obtained from Difco. Iron (III) chloride (FeCl_3_), thiamine hydrochloride, D-(+)-glucose, piperacillin, tazobactam, *n*-dodecyl-β-D-maltoside (DDM), GTP disodium salt, and GDP sodium salt were obtained from Sigma. Luria-Bertani (LB) media was obtained from Millipore. Ciprofloxacin was obtained from Fluka, cefsulodin sodium salt and mecillinam were obtained from RPI, aztreonam was obtained from Alfa Aesar, and cefepime hydrochloride was obtained from Toku-E. Tris-acetate-EDTA (TAE) buffer was obtained from Thermo Scientific, dimethyl sulfoxide (DMSO) was obtained from Fisher, and phosphate buffered saline (PBS) was obtained from Lonza.

### Antibacterial assays

Broth microdilution assays of RUP2 antibacterial activity against wild-type (WT) *S. aureus* NR-46234 (MRSA), mutant *S. aureus* NR-46234 expressing either G196S or N263K mutant FtsZ, *B. subtilis* 23857, *S. saprophyticus* 15305, *L. monocytogenes* 19115, *A. baumannii* 19606, *K. pneumoniae* 13883, *P. aeruginosa* 27853, and *E. coli* 25922 were conducted by preparing two-fold serial dilutions of RUP2 on microtiter plates containing either CAMH media (for all strains except *L. monocytogenes*) or BHI media (for *L. monocytogenes*). Concentrations of RUP2 ranged from 0.16 to 80 µM, with each test concentration being present in triplicate. Log-phase bacteria were added to the plates at a final inoculum of 5 x 10^5^ CFU/mL and the final volume in each well was 100 µL. All plates were incubated with shaking at 37 °C for 18 h, whereupon the bacterial growth in each well was determined by measuring the optical density at 600 nm (OD_600_). The percent bacterial growth in each well was determined by normalizing the corresponding OD_600_ relative to that observed in the absence of test compound (100% growth) and the background value observed for media alone (0% growth).

Broth microdilution assays comparing the antibacterial activities of RUP7, RUP4, and RUP2 against *A. baumannii* 19606 in M9 media were conducted and percent growth determined as described above. Concentrations of each compound ranged from 0.16 to 80 µM, with each test concentration being present in triplicate.

For assays measuring the comparative antibacterial activity of RUP7, RUP4, and RUP2 against a library of *A. baumannii* clinical isolates, six microtiter wells each of DMSO vehicle, 20 µM RUP7, 20 µM RUP4, and 20 µM RUP2 were prepared in M9 media for each isolate tested. The final volume in each well was 100 µL. To all experimental wells, 5 x 10^5^ CFU/mL of exponential bacterial cells were added. The microtiter plates were then incubated with shaking at 37 °C for 18 h. All experimental wells were then serially diluted 10-fold in PBS, and 100 µL of each serial dilution was spread onto duplicate TSA plates. The plates were incubated overnight at 37 °C, and the resulting colonies were counted to determine the bacterial growth (CFU/mL) in each vehicle and compound sample.

To determine the impact of added exogenous Fe^3+^ on the antibacterial activity of RUP7 against *A. baumannii* 19606, serial dilutions of RUP7 were prepared in triplicate as described above in M9 media supplemented with 0, 0.5, 1, 5, or 10 µM of FeCl_3_. The experimental and positive control wells were then inoculated with 5 x 10^5^ CFU/mL of exponential *A. baumannii* 19606 cells grown in the corresponding concentration of FeCl_3_. Plates were incubated and percent growth was determined as described above.

### Cellular uptake assays

For assays comparing the cellular uptake of RUP2 into the Gram-negative strains *A. baumannii* 19606 and *K. pneumoniae* 13883 versus the Gram-positive strains *S. aureus* NR-46234 (MRSA) and *S. saprophyticus* 15305, overnight cultures were diluted 1:100 into 100 mL of CAMH media and incubated with shaking at 37 °C until the OD_600_ reached 0.4–0.8 (approximately 3–4 hours). Each 100 mL culture was centrifuged at 4,000 x *g* for 5 minutes, and the resulting pellets were resuspended in 5 mL of CAMH media and transferred to 14 mL culture tubes. 20 µM of RUP2 was added to the cultures, each being run in quintuplicate. All tubes were incubated with shaking at 37 °C for 30 min. 100 µL of each sample was then serially diluted 10-fold in PBS and 100 µL of each serial dilution was plated on duplicate TSA plates. The plates were incubated overnight at 37 °C, and the colonies on each plate were counted to determine the total CFUs in each sample culture. The remaining 4.9 mL of each culture was centrifuged at 16,000 x *g* for 1–5 min and the resulting pellets were washed with 4 x 1 mL of PBS. After the final centrifugation, each pellet was resuspended in 500 µL of PBS for *A. baumannii* and *K. pneumoniae* cells or PBS supplemented with 1 mg/mL lysostaphin for *S. aureus* and *S. saprophyticus* cells. The *S. aureus* and *S. saprophyticus* cells were then incubated for 1 h at 37 °C. All cells were then ultrasonicated in a 0 °C ice bath for 20 minutes with a 10 second on/off cycle at 30% amplitude using a Qsonica Q500 sonicator equipped with a 0.5-inch probe. The resulting lysates were centrifuged at 16,000 x *g* for 1 minute, and 200 µL of the resulting supernatants were transferred into microfuge tubes. 20 µL of 10x TURBO DNase buffer and 3 µL of TURBO DNase (Invitrogen) were then added to each lysate. The mixtures were then vortexed and incubated at 37 °C for 30 min. After incubation, 220 µL of acetonitrile acidified with 0.1% TFA was added to each sample, and the samples were vortexed at maximum speed for 20 sec. The samples were then centrifuged at 16,000 x *g* for 1 minute, and the concentration of RUP2 in each of the resulting supernatants was characterized by reverse-phase HPLC using standard curves generated for 0.5–4 µM and 4–20 µM RUP2 in DMSO. Differences in the cellular uptake of RUP2 in the Gram-positive versus the Gram-negative strains were compared statistically via one-way ANOVA.

For assays comparing the uptake of RUP7, RUP4, and RUP2 into *A. baumannii* 19606 cells, overnight cultures were diluted 1:100 into 100 mL of M9 media and incubated with shaking at 37 °C until the OD_600_ reached 0.4–0.8 (approximately 4 hours). Each 100 mL culture was centrifuged at 4,000 x *g* for 5 minutes, and the resulting pellets were resuspended in 5 mL of M9 media and transferred to 14 mL culture tubes. 20 µM of RUP7, RUP4, or RUP2 was added to the cultures, with each being run in quintuplicate. All tubes were incubated with shaking at 37 °C for 30 min. The samples were then treated as described above for the *A. baumannii* cultures, with the concentrations of each compound in the final supernatants being characterized by reverse-phase HPLC using standard curves generated for 10–50 µM RUP7, 1–8 µM RUP4, and 0.5–4 µM RUP2 in DMSO. HPLC chromatograms of representative lysate replicates after treatment with each compound are shown in S1–S3 Figs. Reference HPLC chromatograms and associated standard curves used for determination of compound concentrations in lysate replicates are also shown in S1–S3 Figs. Differences in the cellular uptake of RUP7, RUP4, and RUP2 were compared statistically via one-way ANOVA.

HPLC measurements for the cellular uptake assays were performed on a Shimadzu LC-AT liquid chromatograph equipped with a Shimadzu SPD-20AV UV/Vis detector that was set at 260 nm. The samples were run through a 150 x 4.6 mm^2^ SPHER-100 C18 column with a particle size of 5 µm and a pore size of 100 Å (Princeton Chromatography). The samples were injected into the column at a volume of 1 µL and a flow rate of 1 mL/min was applied. The mobile phase was a 10–90% gradient of acidified acetonitrile (0.1% TFA) and water. The total run time for each acquisition was 23 minutes, and the sampling frequency was 2 Hz with a response time of 1 sec. All peak areas were measured using the Shimadzu LabSolutions Lite software package (v5.82).

### Fe^3+^ coordination assay

A solution of 10 µM RUP7 was prepared in methanol and transferred to a quartz cuvette (Hellma) with a 1-cm pathlength in both the excitation and emission directions. The fluorescence emission spectrum of the solution was acquired at 25 °C from 540 to 330 nm, with the excitation wavelength set at 318 nm. FeCl_3_ was then titrated into the solution at concentrations ranging from 1 to 14 µM. After each addition, the sample was allowed to equilibrate at 25 °C for 3 min, whereupon the emission spectrum was reacquired. Each emission spectrum was corrected by subtracting the corresponding emission spectrum of methanol alone run as a background control. The fluorescence measurements were acquired on an AVIV Model ATF105 spectrofluorometer equipped with a thermoelectrically controlled cell holder. The bandwidths in both the excitation and emission directions were set at 5 nm. All spectra were acquired in 1 nm increments, with a 1 sec averaging time at each wavelength reading.

Values of fluorescence emission intensity (*I*) at 418 nm were plotted as a function of Fe^3+^ concentration, with the resulting plot being fit by non-linear least squares regression using the following dimeric binding formalism:


$$I=I_{\text{0}}+\left(\frac{I_{\infty }-I_{\text{0}}}{\text{2}{\left[{RUP\text{7}}_{\text{2}}\right]}_{tot}}\right)\times \left({\left[{RUP\text{7}}_{\text{2}}\right]}_{tot}+{\left[{Fe}^{\text{3}+}\right]}_{tot}+K_{d}\right)-\;\sqrt{{\left({\left[{RUP\text{7}}_{\text{2}}\right]}_{tot}+{\left[{Fe}^{\text{3}+}\right]}_{tot}\;{+K}_{d}\right)}^{\text{2}}-\text{4}{\left[{RUP\text{7}}_{\text{2}}\right]}_{tot}{\left[{Fe}^{\text{3}+}\right]}_{tot}}$$ (1)

In this relationship, *I*_0_ is the fluorescence emission intensity in the absence of Fe^3+^, *I*_∞_ is the fluorescence emission intensity in the presence of an infinite concentration of Fe^3+^, [*RUP7*_*2*_]_*tot*_ is the total RUP7 dimer concentration, [*Fe*^*3+*^]_*tot*_ is the total Fe^3+^ concentration, and *K*_*d*_ is the dissociation constant for the coordination reaction.

### Assays for expression of acinetobactin uptake transporter, exporter, and biosynthesis genes in *A. baumannii*

We determined the expression of acinetobactin uptake transporter genes *bauA*, *bauB*, *bauC*, *bauD*, and *bauE*, the acinetobactin exporter genes *barA* and *barB*, and the acinetobactin biosynthesis genes *basB* and *basD* in *A. baumannii* 19606 cells grown in CAMH media, M9 media, or M9 media supplemented with 10 µM FeCl_3_ by reverse transcription-quantitative polymerase chain reaction (RT-qPCR) using a protocol we have described previously [27]. The primers used for each gene are listed in S1 Table.

### Cloning, expression, and purification of the *A. baumannii* BauA and FtsZ proteins

Cloning, expression, and purification of *A. baumannii* BauA were performed as previously described [51]. Briefly, isolated outer membrane pellets were solubilized with 7% *n*-octylpolyoxyethylene (octyl-POE). The detergent was then exchanged to *n*-dodecyl-β-D-maltoside (DDM) at 3 x the critical micelle concentration (CMC) during the wash step of the Ni^2+^-NTA chromatography. The His-tag was subsequently cleaved by tobacco etch virus (TEV) protease, and the protein was further purified by a second immobilized metal affinity chromatography (IMAC) column. Lastly, the protein was then separated via gel filtration chromatography using Superdex S200 resin in 10 mM Tris-HCl (pH 8.0), 150 mM NaCl, 0.24 mM DDM. *A. baumannii* FtsZ (AbFtsZ) was cloned, expressed, and purified as detailed previously [42].

### Fluorescence anisotropy assays of compound binding to BauA and AbFtsZ

Fluorescence anisotropy measurements were conducted at 25 °C on an AVIV model ATF105 spectrofluorometer equipped with a thermoelectrically controlled cell holder and computer controlled Glan-Thompson polarizers in both the excitation and emission directions. In all experiments, excitation and emission wavelengths were specifically chosen to maximize compound fluorescence and minimize any potential contributions from intrinsic protein fluorescence.

#### Binding of the RUP7-ferric iron coordination complex to BauA.

A solution of 1 µM RUP7 and 1 µM FeCl_3_ (an FeCl_3_ concentration chosen to ensure an excess of Fe^3+^ given the observed 2RUP7:1Fe^3+^ coordination stoichiometry) was prepared in buffer containing 10 mM Tris-HCl (pH 8.0), 150 mM NaCl, and 0.24 mM DDM. The solution was transferred to a quartz ultra-micro cuvette (Hellma) with a 2 x 5 mm aperture and a 15 mm center height. The pathlengths in the excitation and emissions directions were 1 and 0.2 cm, respectively. The fluorescence anisotropy was then recorded as an average of 5 readings, with the excitation and emission wavelengths set at 341 and 457 nm, respectively. The bandwidths were set at 8 nm in both the excitation and emission directions. BauA was then titrated into the solution at concentrations ranging from 0.25 to 2.75 µM. After each addition, the sample was allowed to equilibrate for 5 min, whereupon the fluorescence anisotropy was reacquired in quintuplicate.

Values of fluorescence anisotropy (*r*) were plotted as a function of BauA concentration, with the resulting plot being fit by non-linear least squares regression using the following 1:1 binding formalism:


$$\begin{array}{cc} = & \;r_{\text{0}}+\left(\frac{r_{\infty }-r_{\text{0}}}{\text{2}{{[RUP\text{7}}_{\text{2}}-{Fe}^{\text{3}+}]}_{tot}}\right)\times \left({{[RUP\text{7}}_{\text{2}}-{Fe}^{\text{3}+}]}_{tot}+[BauA{]}_{tot}+K_{d}\right)\\ & -\;\sqrt{{\left({\left[{RUP\text{7}}_{\text{2}}-{Fe}^{\text{3}+}\right]}_{tot}+{\left[BauA\right]}_{tot}\;{+K}_{d}\right)}^{\text{2}}-\text{4}{\left[{RUP\text{7}}_{\text{2}}-{Fe}^{\text{3}+}\right]}_{tot}{\left[BauA\right]}_{tot}} \end{array}$$ (2)

In this relationship, *r*_*0*_ is the fluorescence anisotropy intensity in the absence of BauA, *r*_*∞*_ is the fluorescence anisotropy in the presence of an infinite concentration of BauA, [*RUP7*_*2*_*-Fe*^*3+*^]_*tot*_ is the total concentration of RUP7_2_-Fe^3+^ coordination complex, [*BauA*]_*tot*_ is the total BauA concentration, and *K*_*d*_ is the dissociation constant for the binding reaction.

#### Binding of RUP7 and RUP2 to AbFtsZ.

All the anisotropy studies of AbFtsZ binding were conducted in buffer containing 50 mM Tris-HCl (pH 7.6) and 50 mM KCl. In these characterizations, AbFtsZ was titrated into a solution of compound. After each protein addition, samples were allowed to equilibrate for 5 min, whereupon the fluorescence anisotropy was acquired in quintuplicate.

For our characterization of the RUP7-AbFtsZ binding interaction, the protein was titrated into a solution of 5 µM RUP7, with the final AbFtsZ concentrations ranging from 0 to 62 µM. These anisotropy measurements were conducted using the same ultra-micro cuvette described above in the BauA binding studies. The bandwidths were set to 5 nm in both the excitation and emission directions, and the excitation and emission wavelengths set to 325 and 517 nm, respectively. For our characterization of the RUP2-AbFtsZ binding interaction, the protein was titrated into a solution of 1 µM RUP2, with the final AbFtsZ concentrations ranging from 0 to 3 µM. The bandwidths were set to 7 nm in both the excitation and emission directions, and the excitation and emission wavelengths were set to 300 and 370 nm, respectively. These measurements were conducted using a quartz cuvette with a pathlength of 1 cm in both the excitation and emission directions.

Values of *r* were plotted as a function of AbFtsZ concentration, with the resulting plot being fit by non-linear least squares regression using the following 1:1 binding formalism:


$$r=r_{\text{0}}+\left(\frac{r_{\infty }-r_{\text{0}}}{\text{2}[Comp{]}_{tot}}\right)\times \left([Comp{]}_{tot}+[AbFtsZ{]}_{tot}+K_{d}\right)-\;\sqrt{{\left({\left[Comp\right]}_{tot}+{\left[AbFtsZ\right]}_{tot}\;{+K}_{d}\right)}^{\text{2}}-\text{4}{\left[Comp\right]}_{tot}{\left[AbFtsZ\right]}_{tot}}$$ (3)

In this relationship, *r*_*0*_ is the fluorescence anisotropy intensity in the absence of AbFtsZ, *r*_*∞*_ is the fluorescence anisotropy in the presence of an infinite concentration of AbFtsZ, [*Comp*]_*tot*_ is the total compound concentration, [Ab*FtsZ*]_*tot*_ is the total AbFtsZ concentration, and *K*_*d*_ is the dissociation constant for the binding reaction.

### AbFtsZ polymerization assay

AbFtsZ polymerization was monitored using a 90°-angle light scattering assay conducted at 25 °C on an AVIV Model ATF105 spectrofluorometer equipped with a thermoelectrically controlled cell holder. In this assay, second-order light scattering was measured at an emission wavelength of 680 nm, with the excitation wavelength set at 340 nm. The bandwidth was set at 2 nm in both the excitation and emission directions. Samples containing 250 µM GTP and either DMSO vehicle, 20 µM RUP7, or 40 µM RUP7 were prepared in buffer consisting of 50 mM Tris-HCl (pH 7.6), 50 mM KCl, and 5 mM Mg(CH_3_COO)_2_. The polymerization reaction in each sample was initiated by subsequent addition 10 µM AbFtsZ. The second-order light scattering of each sample was then recorded once per second over a period of 480 seconds, with a time constant of 0.5 seconds. As a negative control, a DMSO vehicle sample was prepared and monitored as described above except that 250 µM GDP was used in place of the GTP. All the light scattering measurements were conducted using the same ultra-micro cuvette described above in the BauA binding studies.

### Antibacterial synergy assays

Studies measuring the antibacterial activity of sub-inhibitory concentrations of RUP7 against *A. baumannii* 19606 when combined with sub-inhibitory concentrations of different clinical antibiotics were performed in microtiter plates. Three replicates each of DMSO vehicle, 80 µM RUP7, 2.5 µM ciprofloxacin, 20 µM cefepime, 20 µM TXH11106, 2560 µM mecillinam, 10 µM ceftazidime, 20 µM piperacillin:tazobactam (8:1), 80 µM aztreonam, 80 µM cefsulodin, or the combination of each antibiotic with RUP7 were prepared in 100 µL of M9 media. Log-phase *A. baumannii* 19606 cells were added to all experimental wells at an inoculum of 1 x 10^6^ CFU/mL. The microtiter plates were then incubated with shaking at 37 °C for 24 hours. Experimental wells were then serially diluted 10-fold in PBS, and 100 µL of each serial dilution was plated on duplicate TSA plates. The plates were incubated at 37 °C overnight, and colonies were counted to quantify bacterial growth (CFU/mL).

The observed logs of cell kill resulting from each combination treatment that exceeded the additive effect from treatment with each agent alone was determined using the following relationship:


$$\text{Log Kill Exceeding Additivity }(\text{LKEA})={\Delta log\left(\frac{CFU}{mL}\right)}_{Comb}-\left[{\Delta log\left(\frac{CFU}{mL}\right)}_{Antib}+{\Delta log\left(\frac{CFU}{mL}\right)}_{RUP\text{7}}\right]$$ (4)

In this relationship, Δ*log*(*CFU/mL*)_*Antib*_, Δ*log*(*CFU/mL*)_*RUP7*_, and Δ*log*(*CFU/mL*)_*Comb*_, were derived by subtraction of the *log*(*CFU/mL*) value for the vehicle-treated culture from the *log*(*CFU/mL*) values for the cultures treated with antibiotic alone, RUP7 alone, and the RUP7-antibiotic combination, respectively. An LKEA >2 was viewed as reflecting synergy.

### Cytotoxicity assay

Cytotoxicity studies of RUP7 against mammalian opossum Kidney (OK) cells (ATCC CRL-1840) were performed using a 4-day MTT assay as described previously [52]. OK cells were treated with six replicates each of DMSO vehicle or RUP7 at a concentration of 5, 10, 20, or 40 µM for 4 days and cell viability was then measured. 4 µM cycloheximide (CHX) was used as a positive control. Statistical comparisons of each treatment relative to DMSO vehicle were performed via one-way ANOVA.

## Results and discussion

### Non-conjugated RUP2 exhibits potent activity against Gram-positive bacteria but poor activity against Gram-negative pathogens

**Fig 2:**
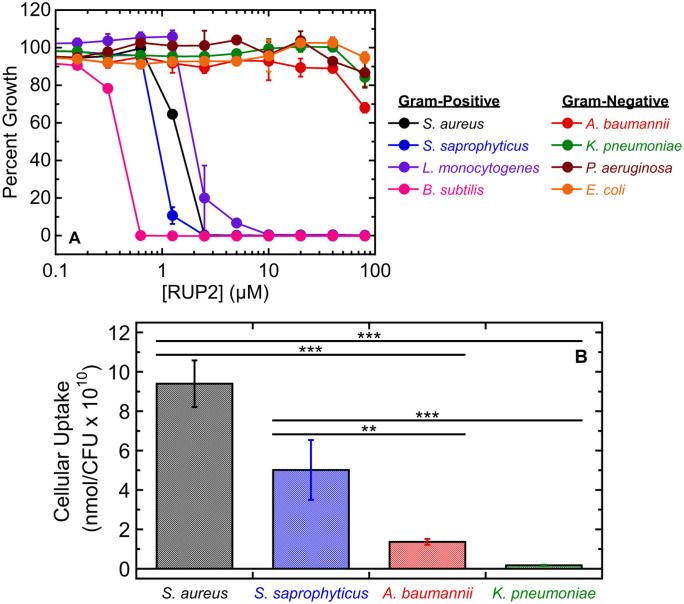
Antibacterial activity and cellular uptake of RUP2 in Gram-positive versus Gram-negative strains. (A) Antibacterial activity of RUP2 against the Gram-positive bacterial strains *S. aureus* NR-46234 (MRSA), *B. subtilis* 23857, *S. saprophyticus* 15305, and *L. monocytogenes* 19115 as well as against the Gram-negative strains *A. baumannii* 19606, *K. pneumoniae* 13883, *P. aeruginosa* 27853, and *E. coli* 25922. All bacterial strains were assayed in CAMH media, except for *L. monocytogenes*, which was assayed in BHI media. Each experimental datapoint represents the average of three replicates, with the error bars reflecting the standard deviations from the mean. (B) Cellular uptake of RUP2 into *S. aureus* NR-46234 (MRSA), *S. saprophyticus* 15305, *A. baumannii* 19606, and *K. pneumoniae* 13883 cells. Each bar represents an average of five replicates, with the error bars reflecting the standard errors from the mean. Statistical comparisons of RUP2 uptake into the Gram-positive relative to the Gram-negative pathogens were conducted via one-way ANOVA. ***, P ≤ 0.001; **, 0.01 ≥ P > 0.001.

We initially sought to delineate the general specificity of antibacterial action associated with non-conjugated RUP2. To this end, we evaluated the antibacterial activity of RUP2 against a broad range of both Gram-positive and Gram-negative bacterial species. Gram-positive species included *S. aureus* NR-46234 (MRSA), *S. saprophyticus* 15305, *L. monocytogenes* 19115, and *B. subtilis* 23857, while Gram-negative species included *A. baumannii* 19606, *K. pneumoniae* 13883, *P. aeruginosa* 27853, and *E. coli* 25922. As revealed by the percent growth profiles shown in Fig 2A (acquired in the presence of RUP2 concentrations ranging from 0.16 to 80 µM), RUP2 exhibits potent activity versus the Gram-positive bacterial species but poor activity versus the Gram-negative species. Control studies showing reduced activity of RUP2 against *S. aureus* NR-46234 expressing G196S or N263K mutant versus wild-type FtsZ are consistent with a FtsZ-targeting mechanism of antibacterial action (S4 Fig). The Gram-positive specificity of antibacterial action exhibited by RUP2 is commonly observed among benzamide-based FtsZ inhibitors [9–23].

### The comparatively poor activity of RUP2 against Gram-negative versus Gram-positive species is directly correlated with correspondingly diminished cellular uptake

To determine whether differential influx may be a contributing factor in the antibacterial specificity of action exhibited by RUP2, we used an HPLC-based assay to quantify the cellular uptake of RUP2 into a large inoculum (~10^11^) of the Gram-negative strains *A. baumannii* 19606 and *K. pneumoniae* 13883 versus the Gram-positive strains *S. aureus* NR-46234 and *S. saprophyticus* 15305 after 30 minutes of treatment with 20 µM compound. Notably, the uptake of RUP2 was significantly greater into the Gram-positive *S. aureus* and *S. saprophyticus* cells relative to the Gram-negative *A. baumannii* and *K. pneumoniae* cells, with measured intracellular quantities of 9.4 ± 1.2 x 10^10^ nmol/CFU for *S. aureus* and 5.0 ± 1.5 x 10^10^ nmol/CFU for *S. saprophyticus* relative to 1.4 ± 0.1 x 10^10^ nmol/CFU for *A. baumannii* and 0.17 ± 0.03 x 10^10^ nmol/CFU for *K. pneumoniae* (Fig 2B). The diminished uptake of RUP2 into the Gram-negative versus Gram-positive bacterial cells correlates well with the comparatively poor activity of the compound against the Gram-negative versus Gram-positive species. In a broader sense, the correlation we observe in the antibacterial and cellular uptake properties of RUP2 highlights differential cellular influx as a contributing factor in the Gram-positive specificity of antibacterial action typically associated with benzamide-based FtsZ inhibitors.

### RUP7 exhibits enhanced activity against *A. baumannii* relative to that of the synthetic chlorocatechol conjugate RUP4 and the non-conjugated RUP2

**Fig 3:**
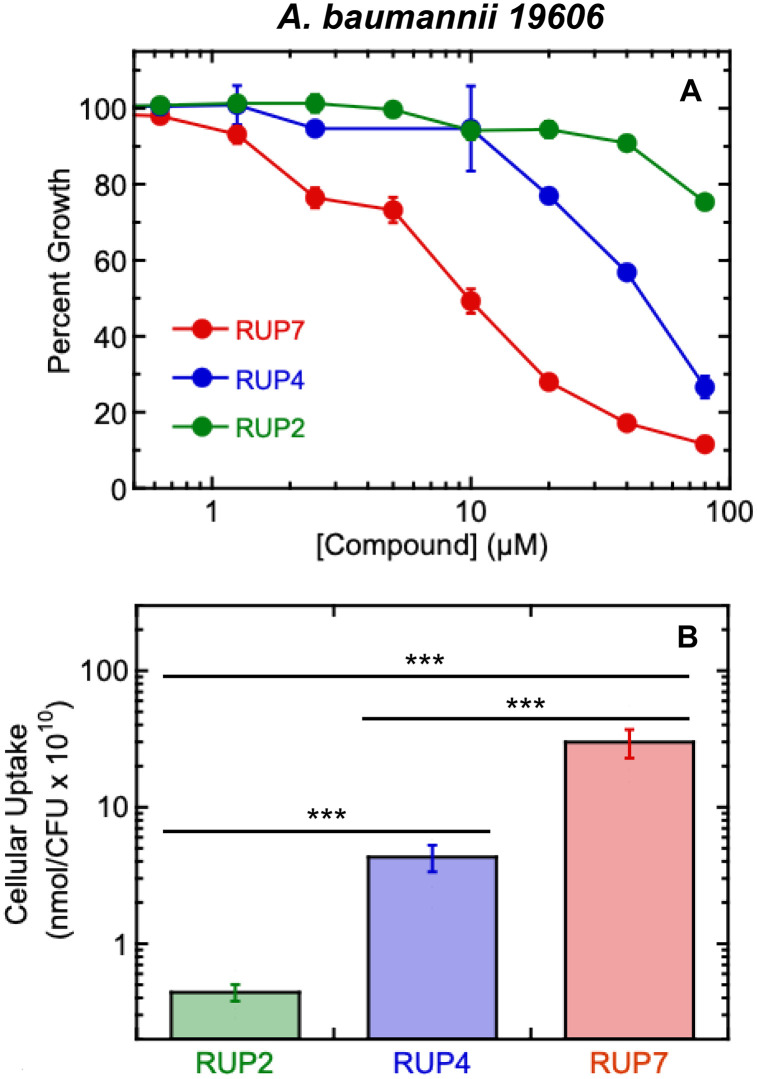
Antibacterial activity and cellular uptake of RUP7, RUP4, and RUP2 in *A. baumannii* 19606. Each experimental datapoint represents the average of three replicates, with the error bars reflecting the standard deviations from the mean. (B) Cellular uptake of RUP7, RUP4, and RUP2 into *A. baumannii* 19606 cells. Each bar represents the average of five replicates, with the error bars reflecting the standard errors from the mean. Statistical comparisons of compound uptake were conducted via one-way ANOVA. ***, P ≤ 0.001.

The rational design of RUP7 was motivated by the hypothesis that conjugating the native siderophore acinetobactin to the oxazole-benzamide FtsZ inhibitor would enhance the activity against *A. baumannii* relative to the non-conjugated RUP2 as well as the synthetic chlorocatechol siderophore conjugate RUP4. We first tested this hypothesis against *A. baumannii* 19606 by measuring percent growth in Fe^3+^-limiting M9 media at RUP7, RUP4, and RUP2 concentrations ranging from 0.16 to 80 µM. Consistent with our hypothesis, the activity of RUP7 was enhanced over a broad concentration range relative to both RUP4 and RUP2 (Fig 3A). RUP4 had modest activity at higher concentration levels, with RUP2 exhibiting minimal activity even at the highest concentration tested. Thus, conjugation of the native acinetobactin siderophore to the FtsZ inhibitor markedly enhanced activity against *A. baumannii*, with this enhancement being significantly greater than that produced by conjugation to the synthetic chlorocatechol siderophore.

### The enhanced activity of RUP7 against *A. baumannii* relative to RUP4 and RUP2 is correlated with a correspondingly enhanced cellular uptake

To explore the basis for the enhanced activity of RUP7 against *A. baumannii* relative to RUP4 and RUP2, we compared the uptake of the three compounds into *A. baumannii* 19606 cells using the same HPLC-based assay described above. Significantly, the relative cellular uptake of the compounds correlated with their relative antibacterial activities. In this connection, RUP7 exhibited the greatest extent of cellular uptake at 29.9 ± 15.8 x 10^10^ nmol/CFU, followed by RUP4 at 4.3 ± 2.1 x 10^10^ nmol/CFU, and RUP2 at 0.44 ± 0.14 x 10^10^ nmol/CFU (Fig 3B). These findings provide a clear demonstration that siderophore conjugation enhances the cellular uptake of the FtsZ inhibitor into *A. baumannii* cells, with conjugation to acinetobactin yielding the greatest degree of uptake.

### The enhanced activity of RUP7 relative to RUP4 and RUP2 extends to a library of *A. baumannii* clinical isolates

**Fig 4:**
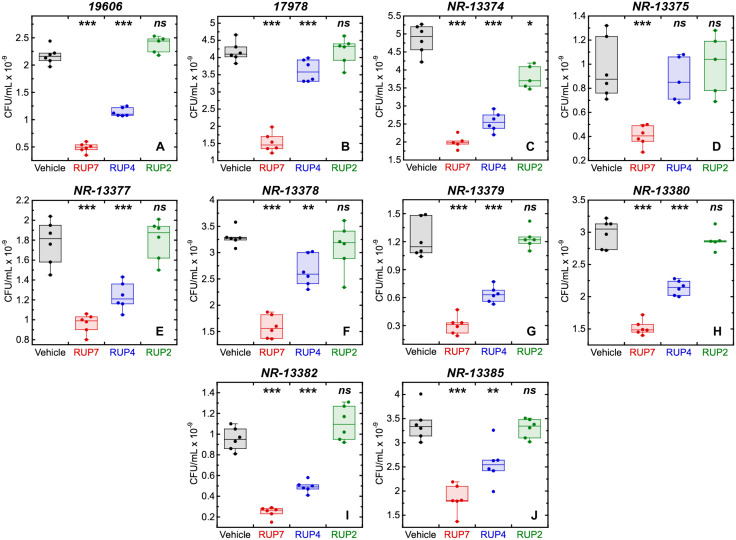
Antibacterial activity of RUP7, RUP4, and RUP2 compared to DMSO vehicle against *A. baumannii* reference strains 19606 (A) and 17978 (B) as well as clinical isolates NR-13374 (C), NR-13375 (D), NR-13377 (E), NR-13378 (F), NR-13379 (G), NR-13380 (H), NR-13382 (I), and NR-13385 (J). Each box plot represents 6 replicates. Statistical comparisons of RUP7, RUP4, or RUP2 relative to DMSO vehicle were conducted via one-way ANOVA. ***, P ≤ 0.001; **, 0.01 ≥ P > 0.001; *, 0.05 ≥ P > 0.01; *ns*, not significant.

Our initial characterization of the relative activities of RUP7, RUP4, and RUP2 was focused on a single *A. baumannii* strain (19606). We also extended these characterizations to include a library of eight additional *A. baumannii* clinical isolates (NR-13374, NR-13375, NR-13377, NR-13378, NR-13379, NR-13380, NR-13382, and NR-13385) as well as reference ATCC strain 17978. In these assays, we compared the activity of RUP7, RUP4, and RUP2 against the different isolates by measuring the number of CFUs after 18 hours of treatment at 20 µM, a concentration at which RUP7 inhibits the growth of *A. baumannii* 19606 by approximately 75% (see Fig 3A). Significantly, the CFU-based results (shown in Fig 4) revealed a similar pattern of relative activity to that observed against 19606, with RUP7 consistently exhibiting the greatest extent of growth inhibition relative to vehicle, followed by RUP4, which had a modest inhibitory effect against most isolates. RUP2 was completely inactive against all but one isolate. Note that two of the isolates tested (NR-13375 and NR-13377) are resistant to the current standard-of-care antibiotics piperacillin:tazobactam and ceftazidime. Thus, the activity of RUP7 also extends to resistant isolates of *A. baumannii*.

### RUP7 coordinates ferric iron (Fe^3+^) with a 2:1 stoichiometry

**Fig 5:**
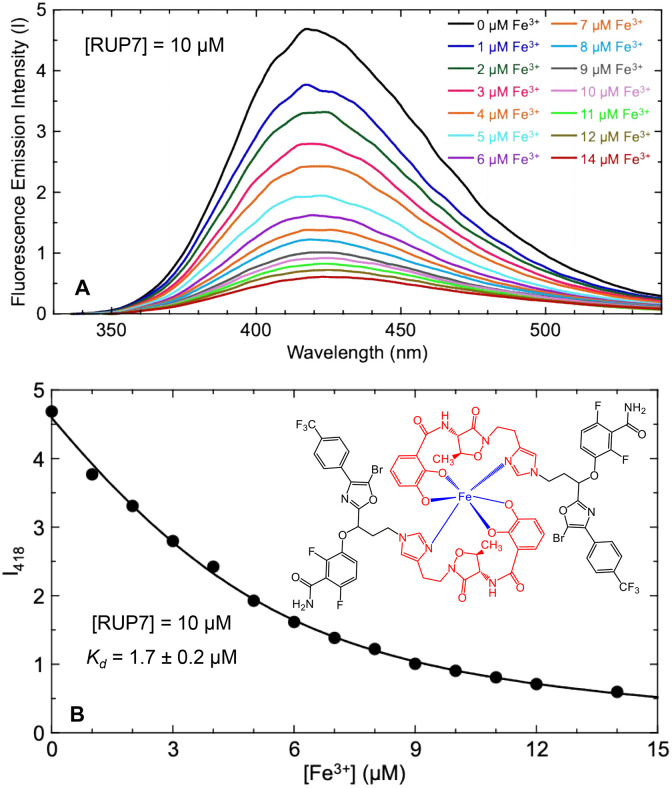
Coordination of ferric iron (Fe^3+^) by RUP7. (B) Values of fluorescence emission intensity at 418 nm (I_418_) plotted as a function of Fe^3+^ concentration. The solid line represents a non-linear least squares fit of the I_418_ values using Equation (1), with the indicated *K*_*d*_ value for the RUP7_2_-Fe^3+^ coordination reaction being derived from this fit. The inset depicts a proposed hexadentate model for the RUP7_2_-Fe^3+^ coordination complex.

As Fe^3+^ coordination is critical for siderophore recognition and intracellular uptake via endogenous siderophore transport pathways, we next endeavored to establish the ability of RUP7 to coordinate Fe^3+^ in a manner analogous to free unconjugated acinetobactin. To this end, we used a fluorescence-based approach to detect and characterize the interaction of RUP7 with Fe^3+^ by leveraging the intrinsic fluorescence properties of the conjugated acinetobactin functionality, which were similar to those previously defined by Kim and coworkers for unconjugated acinetobactin [50]. Addition of Fe^3+^ induced a profound concentration-dependent decrease in the magnitude of the RUP7 fluorescence emission spectrum (Fig 5A), indicative of RUP7 complexation with Fe^3+^. The concentration dependence of the Fe^3+^-induced decrease in RUP7 fluorescence emission intensity at 418 nm (I_418_) could not be fit by a 1:1 binding model but was well fit by a dimeric (2RUP7:1Fe^3+^) binding model, which yielded an apparent *K*_*d*_ of 1.7 ± 0.2 µM for the RUP7_2_-Fe^3+^ coordination reaction (Fig 5B). This observed stoichiometry for Fe^3+^ coordination is consistent with that previously reported by Kim and coworkers for unconjugated acinetobactin [50], a gratifying concordance indicating that conjugation of the FtsZ inhibitor to the imidazole moiety of acinetobactin does not interfere with the ability of the siderophore to coordinate Fe^3+^. Previously reported crystallographic and computational studies with acinetobactin have shown that coordination of Fe^3+^ involves both the imidazole and catechol functionalities of two acinetobactin molecules to form a hexadentate coordination complex [49,51,53]. It is therefore likely that two RUP7 molecules form a similar hexadentate coordination complex with Fe^3+^ (as schematically depicted in the inset of Fig 5B).

### The activity of RUP7 against *A. baumannii* is acutely dependent on the concentration of exogenous Fe^3+^

**Fig 6:**
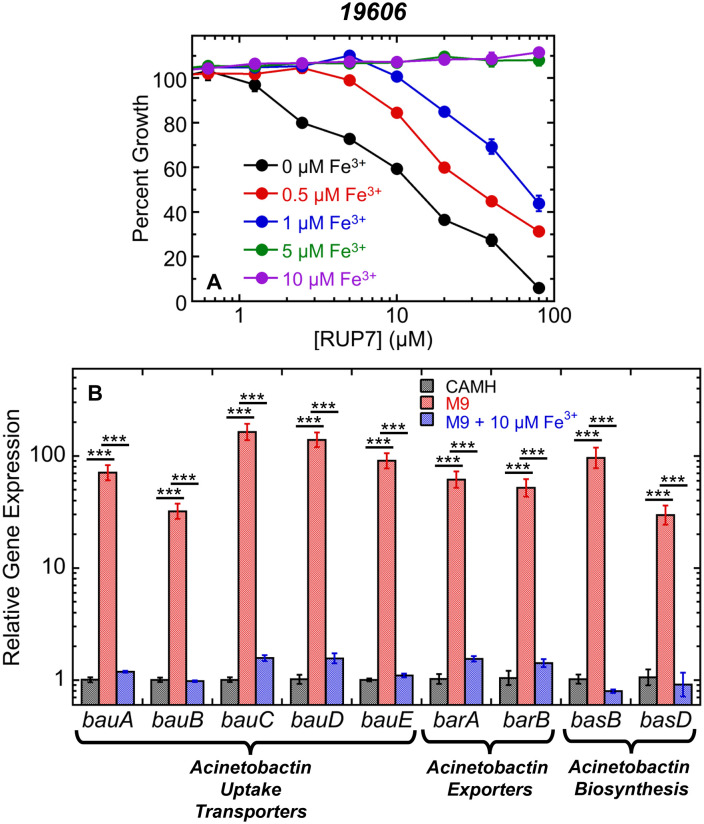
Impact of environmental Fe^3+^ on the activity of RUP7 against *A. baumannii* 19606 and the expression of acinetobactin uptake transporter, exporter, and biosynthesis genes. The concentrations of added exogenous Fe^3+^ are indicated. Each experimental datapoint represents the average of three replicates, with the error bars reflecting the standard deviations from the mean. (B) Expression of acinetobactin uptake transporter, exporter, and biosynthesis genes in *A. baumannii* 19606 cells grown in CAMH media (black), M9 media (red), or M9 media supplemented with 10 µM Fe^3+^ (blue). Each bar represents an average of 5 replicates, with the error bars reflecting the standard errors of the mean. Statistical comparisons were conducted via one-way ANOVA. ***, P ≤ 0.001.

In addition to demonstrating the coordination of Fe^3+^ by RUP7, we wanted to determine if the antibacterial activity of the conjugate depends on the concentration of exogenous Fe^3+^. To this end, we measured the activity of RUP7 (at concentrations ranging from 0.16 to 80 µM) against *A. baumannii* 19606 cells grown in Fe^3+^-limiting M9 media supplemented with either 0, 0.5, 1, 5, or 10 µM exogenous Fe^3+^. RUP7 activity was significantly attenuated in a Fe^3+^-dependent manner, with activity being abolished in the presence of 5 and 10 µM added Fe^3+^, even at the highest tested concentrations of RUP7 (Fig 6A). Thus, the activity of RUP7 is highly dependent on Fe^3+^-limiting environmental conditions.

### Expression of acinetobactin biosynthesis, exporter, and uptake transporter genes in *A. baumannii* is highly upregulated in Fe^3+^-limiting versus Fe^3+^-rich conditions

To better understand the basis for the Fe^3+^ dependence of RUP7 activity, we used RT-qPCR to evaluate the expression of acinetobactin biosynthesis, exporter, and uptake transporter genes by *A. baumannii* 19606 cells in Fe^3+^-rich versus Fe^3+^-limiting growth environments. In these characterizations, we investigated the following acinetobactin uptake transporter genes: the outer membrane transporter *bauA*, the periplasmic chaperone *bauB*, the heterodimeric inner membrane transporter components *bauC* and *bauD*, and the inner membrane transporter ATPase *bauE*. We also investigated the acinetobactin exporter genes *barA* and *barB* as well as the acinetobactin biosynthesis genes *basB* and *basD*, which encode critical enzymes for late stage acinetobactin assembly from precursors. Expression of RNA polymerase component genes *rpoB* and *rpoD* was used as endogenous controls for all comparisons.

All the tested acinetobactin biosynthesis, exporter, and uptake transporter genes were highly upregulated in Fe^3+^-limiting M9 media compared to Fe^3+^-rich CAMH media (Fig 6B). Among the uptake transporter genes, expression of *bauC* was the most upregulated at 163-fold, followed by *bauD*, *bauE*, *bauA*, and *bauB* at 139-, 90.3-, 71.0-, and 32.0-fold, respectively. Expression of the exporter genes *barA* and *barB*, as well as the biosynthesis genes *basB* and *basD*, was upregulated to an extent ranging from 29.7- to 96.2-fold. The upregulated expression under Fe^3+^-limiting conditions we observe for the biosynthesis genes *basB* and *basD* and the inner membrane transporter component gene *bauD* is consistent with that previously reported by Sheldon and Skaar in *A. baumannii* 17978 cells [44]. As expected, the addition of exogenous 10 µM Fe^3+^ to M9 media repressed the expression of all tested genes to a level comparable with that observed in CAMH (Fig 6B). Our gene expression results, coupled with the Fe^3+^ dependence of RUP7 activity, point to upregulated acinetobactin uptake transport systems as being important determinants of RUP7 activity against *A. baumannii*. Importantly, such upregulation would be manifest in the Fe^3+^-limiting conditions typically present at common sites of *A. baumannii* infection, including the respiratory tract, bloodstream, urinary tract, skin, and soft tissue [44,54–56]. It is also important to note that upregulated biosynthesis of endogenous acinetobactin under Fe^3+^-limiting conditions introduces potential competition with RUP7 for transporter-mediated cellular uptake. That said, the activity of RUP7 we observe provides an indication that such potential competition can be overcome.

### The RUP7_2_-Fe^3+^ coordination complex directly interacts with the acinetobactin outer membrane uptake transporter BauA

**Fig 7:**
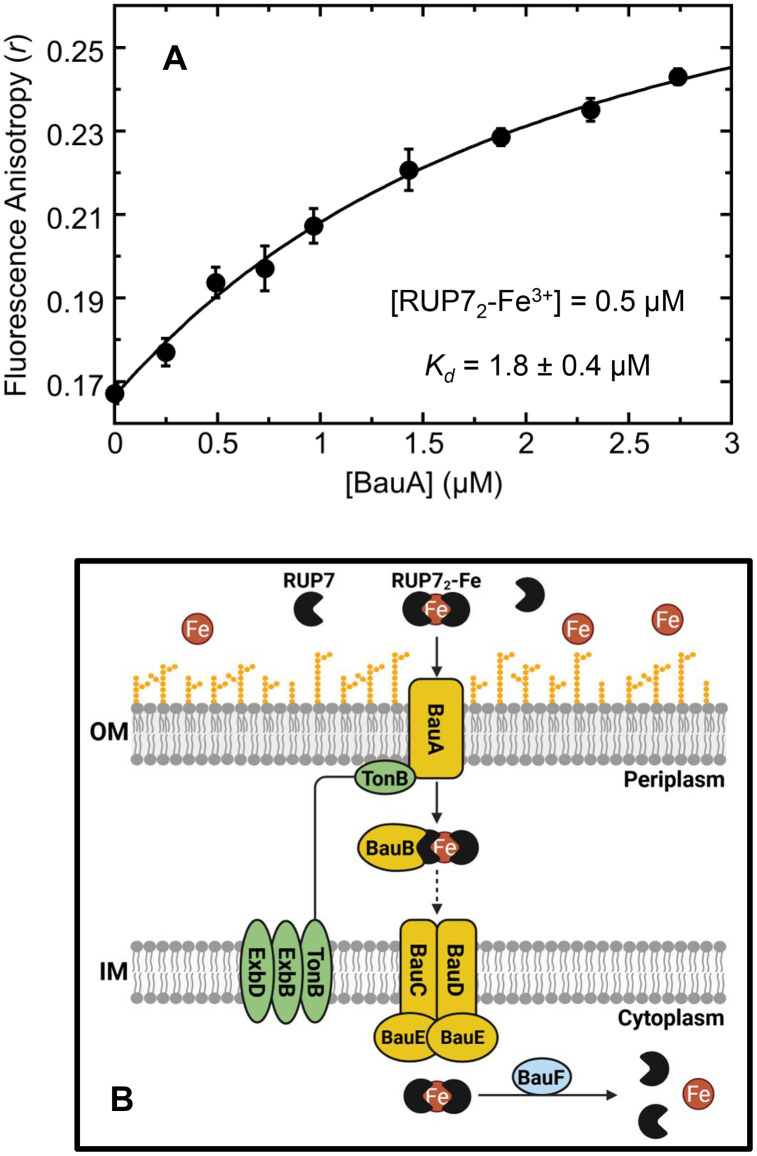
Binding of the RUP7_2_-Fe^3+^ coordination complex to *A. baumannii* BauA and proposed model for the uptake of RUP7_2_-Fe^3+^ via the BauABCDEF acinetobactin uptake transport system. Each experimental datapoint represents the average of five replicates, with the error bars reflecting the standard deviations from the mean. The solid line represents a non-linear least squares fit of the *r* values using Equation (2), with the indicated *K*_*d*_ value for the binding interaction being derived from this fit. **(B)** Proposed model for the uptake of RUP7_2_-Fe^3+^ via the BauABCDEF acinetobactin uptake transport system.

Given that RUP7 coordinates Fe^3+^ in a similar manner to unconjugated acinetobactin, we hypothesized that RUP7 can enter *A. baumannii* cells via the acinetobactin uptake transport system BauABCDEF, which is highly upregulated under the Fe^3+^-limiting conditions that promote RUP7 activity. In this connection, we probed for a direct interaction of the RUP7_2_-Fe^3+^ coordination complex with the acinetobactin outer membrane uptake transporter BauA. To this end, we leveraged the intrinsic fluorescence properties of RUP7 by monitoring the fluorescence anisotropy of the RUP7_2_-Fe^3+^ coordination complex as a function of added BauA. Addition of BauA induced a protein-dependent increase in fluorescence anisotropy (Fig 7A) consistent with a direct binding interaction. The anisotropy increase was well fit by the 1:1 binding formalism described by Equation (2), with this analysis yielding an apparent *K*_*d*_ of 1.8 ± 0.4 µM for the binding of RUP7_2_-Fe^3+^ to BauA. This direct binding interaction suggests that BauA may indeed serve as the outer membrane transporter for uptake of RUP7_2_-Fe^3+^ into *A. baumannii*, as schematically depicted in Fig 7B. Following BauA-mediated transport across the outer membrane, Fe^3+^-charged acinetobactin is then transported across the periplasm and inner membrane via the BauBCDE transporter system [44]. It is therefore possible that RUP7_2_-Fe^3+^ is transported via the same system (Fig 7B). Additional studies with specific Bau mutants will be required to fully validate this proposed uptake pathway.

### RUP7 binds AbFtsZ and inhibits GTP-dependent AbFtsZ polymerization

**Fig 8:**
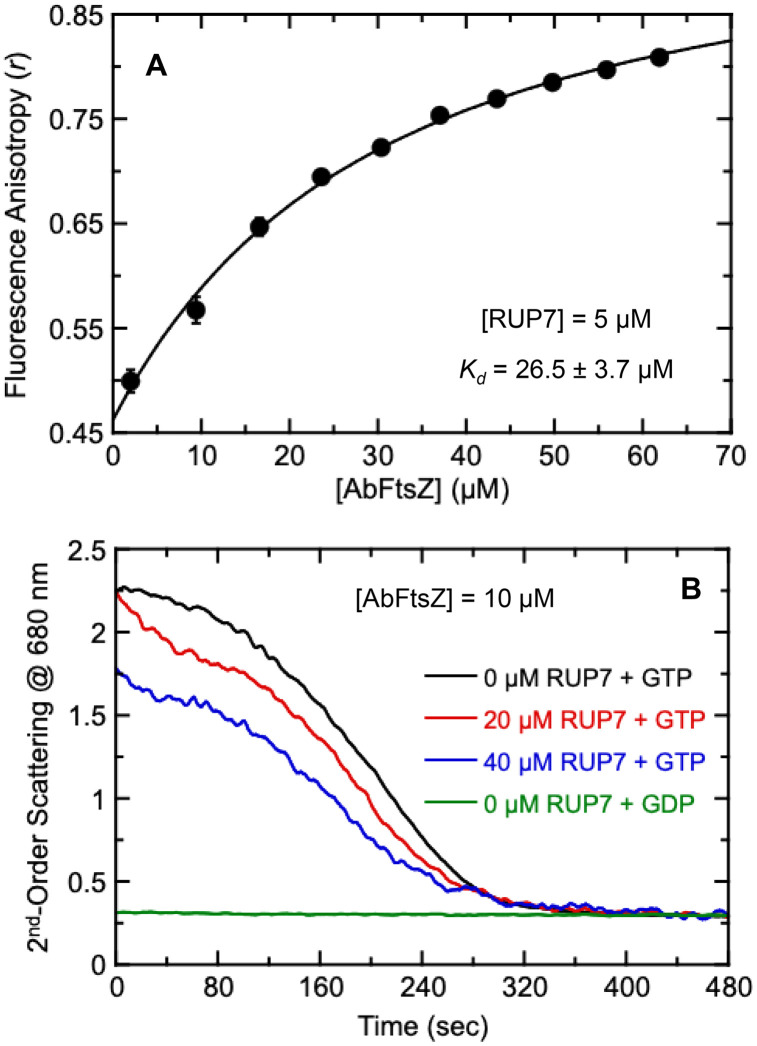
Binding of RUP7 to AbFtsZ and the impact of binding on GTP-dependent AbFtsZ polymerization. Each experimental datapoint represents the average of five replicates, with the error bars reflecting the standard deviations from the mean. The solid line represents a non-linear least squares fit of the *r* values using Equation (3), with the indicated *K*_*d*_ value being derived from this fit. (B) Time-dependent 90°-angle light scattering profiles of 10 µM AbFtsZ in the presence of 0 µM RUP7 + 250 µM GTP (black), 20 µM RUP7 + 250 µM GTP (red), 40 µM RUP7 + 250 µM GTP (blue), or 0 µM RUP7 + 250 µM GDP (green).

We next wanted to confirm that the RUP7-acinetobactin conjugate can target AbFtsZ. To this end, we probed for the direct binding of RUP7 to AbFtsZ using fluorescence anisotropy. Addition of AbFtsZ induced an increase in the fluorescence anisotropy of RUP7 in a concentration-dependent manner (Fig 8A), indicative of a direct binding interaction. Analysis of this anisotropy profile with Equation (3) yielded an affinity constant (*K*_*d*_) of 26.5 ± 3.7 µM. Corresponding anisotropy measurements with non-conjugated RUP2 yielded a *K*_*d*_ of 0.23 ± 0.09 µM (S5 Fig), a binding affinity two orders of magnitude greater than that of RUP7. Thus, conjugation of the bulky acinetobactin moiety markedly reduced affinity for AbFtsZ. Yet, despite this reduced target affinity, the RUP7-acinetobactin conjugate is associated with significantly enhanced activity against *A. baumannii* relative to non-conjugated RUP2, due in large part to its enhanced intracellular uptake. These collective results highlight the contributions of intracellular uptake to antibacterial activity, perhaps even more so than target affinity.

We also explored the impact RUP7 on the GTP-dependent self-polymerization of AbFtsZ using a 90°-angle light scattering assay analogous to that previously established by Mukherjee and Lutkenhaus [57]. In this assay, 250 µM GTP was combined with DMSO vehicle (0 µM RUP7), 20 µM RUP7, or 40 µM RUP7. In addition, a 0 µM RUP7 sample with 250 µM GDP in place of GTP was also prepared as a negative control. 10 µM AbFtsZ was then added to each sample, thereby initiating polymerization in the GTP-containing samples. Immediately following addition of AbFtsZ, 2^nd^-order light scattering was monitored as a function time, with the resulting light scattering profiles being shown in Fig 8B. Note that the GTP-containing samples each exhibit a time-dependent transition from a higher to a lower level of light scattering, reflecting a transition from a polymerized to a depolymerized state as the GTP is exhausted due to hydrolysis [57]. By contrast, no such transition is observed in the GDP-containing sample, which exhibits a steady low level of light scattering indicative of a lack of induced polymerization. Significantly, the presence of 20 and 40 µM RUP7 systematically decreases the maximal light scattering levels in a concentration-dependent manner, indicating correspondingly reduced extents of GTP-dependent polymerization in the presence of increasing compound concentrations. These observations are consistent with RUP7 acting as an inhibitor of AbFtsZ polymerization. Scoffone *et al.* observed a similar behavior associated with the benzothiadiazole AbFtsZ inhibitor C109 [24].

### RUP7 exhibits bactericidal synergy against *A. baumannii* when used in combination with antibiotics that target PBP3

**Fig 9:**
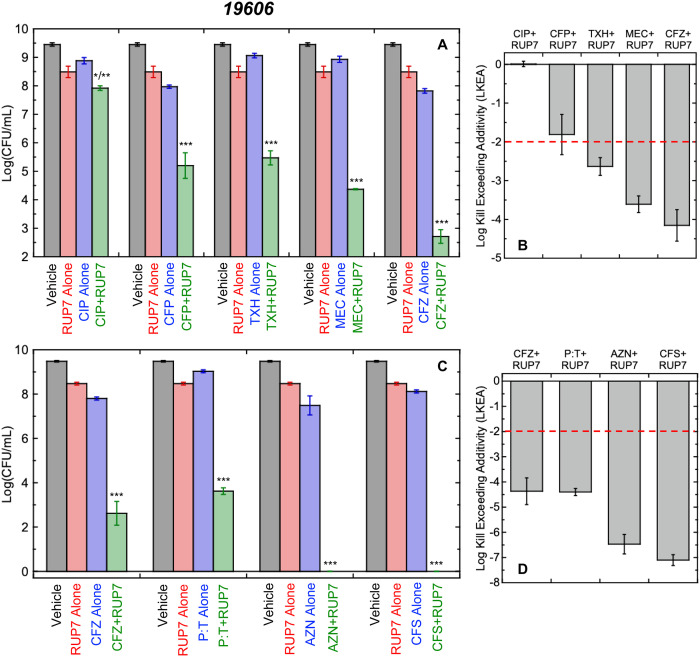
Assessment of bactericidal synergy associated with different antibiotic+RUP7 combinations. (A) Screen for antibacterial synergy of RUP7 in combination with the antibiotics ciprofloxacin (CIP), cefepime (CFP), TXH11106 (TXH), mecillinam (MEC), or ceftazidime (CFZ) against *A. baumannii* 19606 in M9 media. (B) Logs of kill exceeding additivity (LKEA) for each antibiotic+RUP7 combination shown in panel (A), as derived using Equation (4). (C) Antibacterial synergy of RUP7 in combination with the antibiotics ceftazidime (CFZ), piperacillin:tazobactam (8:1) (P:T), aztreonam (AZN), or cefsulodin (CFS) against *A. baumannii* 19606 in M9 media. (D) LKEA for each antibiotic+RUP7 combination shown in panel (C), derived as above. In panels (A) and (C), the bacteria were treated with DMSO vehicle (black), RUP7 alone (red), antibiotic alone (blue), or RUP7 in combination with antibiotic (green). The red dashed line in panels (B) and (D) denotes the 2-log threshold above which LKEA is reflective of synergy. All data bars represent the average of three replicates, with the associated error bars reflecting the standard deviations from the mean. Statistical comparisons of each antibiotic+RUP7 combination relative to either agent alone were conducted via one-way ANOVA. ***, P ≤ 0.001; **, 0.01 ≥ P > 0.001; *, 0.05 ≥ P > 0.01.

Synergistic combinations of antibiotics are an appealing strategy to increase antibacterial potency, reduce cytotoxicity, and mitigate the potential for the emergence of resistance [58–60]. We probed for antibiotics that can act synergistically in combination with RUP7. Toward this end, we combined RUP7 and representative members of different classes of antibiotics, including the DNA gyrase inhibitor ciprofloxacin (CIP), the PBP1a/1b inhibitor cefepime (CFP), the MreB inhibitor TXH11106 (TXH), the PBP2 inhibitor mecillinam (MEC), and the PBP3 inhibitor ceftazidime (CFZ). In each analysis, we tested DMSO vehicle, RUP7 alone, antibiotic alone, or a combination of RUP7 and antibiotic. Upon assessment of the number of CFUs after 24 hours of treatment, the combination of RUP7 with all antibiotics except CIP exhibited notably reduced CFUs relative to DMSO vehicle or test agent alone (Fig 9A). By contrast, all test agents alone exhibited CFUs comparable to that of vehicle. To assess synergy, we further quantified the log kill exceeding additivity (LKEA) for each antibiotic+RUP7 combination relative to both agents alone using Equation (4), with an LKEA of >2-logs being reflective of synergy. Note that the combinations of CFZ+RUP7, MEC+RUP7, and TXH+RUP7 were all associated with bactericidal synergy, with CFZ+RUP7 exhibiting the greatest degree of synergy (LKEA = 4.2 ± 0.4), followed by MEC+RUP7 (LKEA = 3.6 ± 0.2) and TXH+RUP7 (LKEA = 2.6 ± 0.2) (Fig 9B). Neither the combination of CIP+RUP7 (LKEA = 0 ± 0.1) nor CFP+RUP7 (LKEA = 1.8 ± 0.5) was associated with any significant synergistic activity.

Our initial screen revealed that the greatest degree of bactericidal synergy was associated with combination of RUP7 and the PBP3 inhibitor CFZ. We further explored this behavior by performing a second screen with a panel of four PBP3-targeting antibiotics that included piperacillin:tazobactam at a ratio of 8:1 (P:T), aztreonam (AZN), and cefsulodin (CFS) in addition to CFZ. All four combinations were associated with a significant reduction in CFUs relative to vehicle or either test agent alone, with no detectable colonies being observed for either the AZN+RUP7 or CFS+RUP7 combination (Fig 9C). All four antibiotic + RUP7 combinations exhibited significant bactericidal synergy, with CFS+RUP7 and AZN+RUP7 exhibiting the greatest degrees of synergy (LKEA = 7.1 ± 0.2 and 6.5 ± 0.4, respectively), followed by P:T+RUP7 (LKEA = 4.4 ± 0.1) and CFZ+RUP7 (LKEA = 4.4 ± 0.5) (Fig 9D). The synergy was sufficiently significant for the AZN+RUP7 and CFS+RUP7 combinations that we observed complete kill of the entire bacterial inoculum after 24 hours of treatment (Fig 9C).

PBP3 plays an important role in bacterial cell division, serving as a critical component of the bacterial divisome [61,62]. Significantly, the localization of PBP3 to the midcell of a dividing bacterium depends on a functional FtsZ protein [63,64]. In addition, constriction of the FtsZ Z-ring during bacterial cytokinesis relies on cell wall synthesis at midcell, a process for which PBP3 is indispensable [65]. This complementarity of function between PBP3 and FtsZ for cell division could account for the significant synergy we observe with the combination of RUP7 and PBP3 inhibitors. In the aggregate, our synergy results highlight combinations of FtsZ and PBP3 inhibitors as an appealing pharmacologic approach for the treatment of *A. baumannii* infections.

### RUP7 exhibits minimal cytotoxicity against mammalian cells

**Fig 10:**
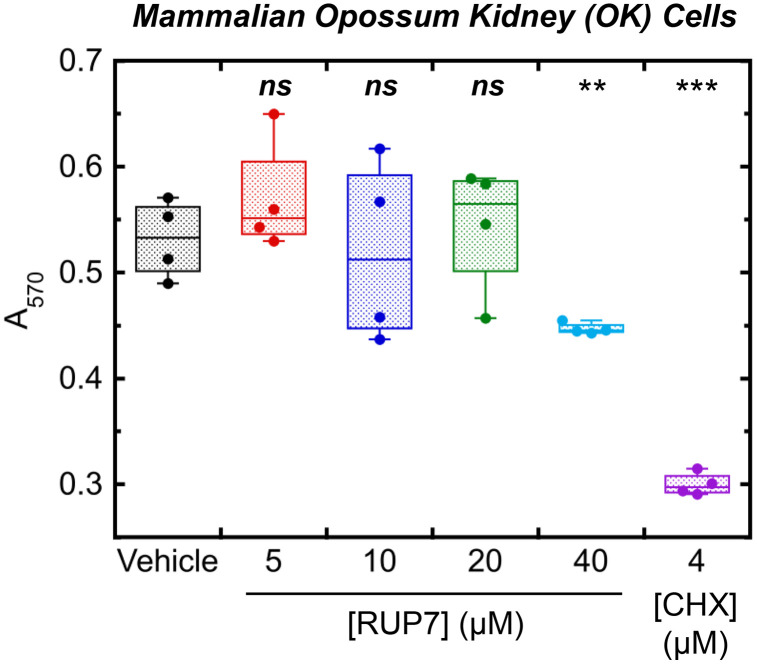
MTT-based assay for cytotoxicity against mammalian opossum kidney (OK) cells associated with exposure to RUP7. Cells were treated with DMSO vehicle (black), 5 µM RUP7 (red), 10 µM RUP7 (blue), 20 µM RUP7 (green), 40 µM RUP7 (cyan), or 4 µM cycloheximide (CHX) (purple). Each box plot represents 4 replicates, and statistical comparisons of each treatment relative to DMSO vehicle were performed via one-way ANOVA. A_570_, absorbance at 570 nm; ***, P ≤ 0.001; **, 0.01 ≥ P ≥ 0.001; *ns*, not significant.

The potential utility of any antibacterial agent requires a specificity of action against bacterial versus host cells. We therefore sought to evaluate RUP7 for any potential cytotoxicity against mammalian cells. To this end, we used a 4-day MTT colorimetric assay to monitor the viability of opossum kidney (OK) cells (ATCC CRL-1840) upon exposure to RUP7 at concentrations ranging from 5 to 40 µM. Additionally, we included DMSO vehicle as a negative control and 4 µM cycloheximide (CHX) as a positive control. No significant reduction in A_570_ was observed at an RUP7 concentration of 5, 10, or 20 µM when compared to vehicle treatment, with 40 µM RUP7 resulting in only a 16% reduction in A_570_ (Fig 10). As expected, 4 µM CHX treatment resulted in a 44% decline in A_570_. While these encouraging initial results reveal minimal mammalian cytotoxicity on the part of RUP7, additional MTT and clonogenic (colony formation) studies against a broader panel of both mammalian and human cells will be required to fully confirm this behavior.

## Conclusions

Here we describe RUP7 as a second-generation benzamide-based FtsZ inhibitor-siderophore conjugate in which the FtsZ inhibitor RUP2 is conjugated to acinetobactin, a principal native siderophore of *A. baumannii*. Conjugation to acinetobactin significantly enhances intracellular uptake and activity against a range of clinical *A. baumannii* isolates relative to non-conjugated RUP2 and our first-generation FtsZ inhibitor-siderophore conjugate RUP4, which contains a synthetic chlorocatechol moiety as its conjugated siderophore in place of acinetobactin. RUP7 coordinates Fe^3+^ in a similar manner to unconjugated acinetobactin, with this coordination being an important determinant of antibacterial activity. The activity of RUP7 is acutely enhanced in Fe^3+^-limiting environments, like those prevalent at common sites of *A. baumannii* infection. RUP7 directly interacts with the BauA outer membrane acinetobactin uptake transporter, and its activity is correlated with an upregulation of the BauABCDEF acinetobactin uptake system in Fe^3+^-limiting conditions. RUP7 is also able to directly target AbFtsZ, albeit with reduced affinity relative to non-conjugated RUP2, whereupon it acts as an inhibitor of GTP-dependent AbFtsZ polymerization. RUP7 exhibits significant bactericidal synergy against *A. baumannii* upon combination with clinical antibiotics that target PBP3, while also exhibiting minimal cytotoxicity against mammalian cells. Viewed as a whole, our results validate siderophore conjugation as a design strategy for enhancing the cellular uptake and activity of benzamide-based FtsZ inhibitors against clinically important Gram-negative bacterial pathogens like *A. baumannii*, while also highlighting the appeal of combining design-optimized FtsZ inhibitor-siderophore conjugates together with PBP3-targeting antibiotics for the treatment of *A. baumannii* infections.

## Supporting information

S1 Appendix Synthesis of RUP7. (PDF)

S1 Fig Representative HPLC chromatograms used for determining RUP7 concentration in *A. baumannii* 19606 lystates. (PDF)

S2 Fig Representative HPLC chromatograms used for determining RUP4 concentration in *A. baumannii* 19606 lystates. (PDF)

S3 Fig Representative HPLC chromatograms used for determining RUP2 concentration in *A. baumannii* 19606 lystates. (PDF)

S4 Fig Antibacterial activity of RUP2 against wild-type *S. aureus* clinical isolate NR-46234 (MRSA) and corresponding mutant strains expressing G196S or N263K mutant FtsZ. (PDF)

S5 Fig Binding of RUP2 to AbFtsZ as reflected by changes in the fluorescence anisotropy (*r*) of RUP2 as a function of increasing concentrations of AbFtsZ. (PDF)

S1 Table Sequences of the oligonucleotide primers used in the RT-qPCR studies of *A. baumannii* 19606 cells grown in CAMH media, M9 media, or M9 media + 10 µM Fe^3+^. (PDF)
