# Supplementary material for: A FtsZ inhibitor-acinetobactin conjugate with enhanced cellular uptake in Acinetobacter baumannii acts synergistically in combination with PBP3-targeting antibiotics
Source: PLoS One. 2025 Oct 14;20(10):e0334409. doi: 10.1371/journal.pone.0334409 (PMC12520410; doi:10.1371/journal.pone.0334409)
Supplement: S1 Appendix — (PDF) [file pone.0334409.s001.pdf]

Appendix S1: Synthesis of RUP7. RUP7 was synthesized according to Scheme S1, with the synthetic routes for intermediates **5** and **9** being based on the approach previously reported by Takeuchi *et al.* [Synthesis of Acinetobactin. Chem Pharm Bull. 2010;58(11):1552-3. doi: 10.1248/cpb.58.1552].

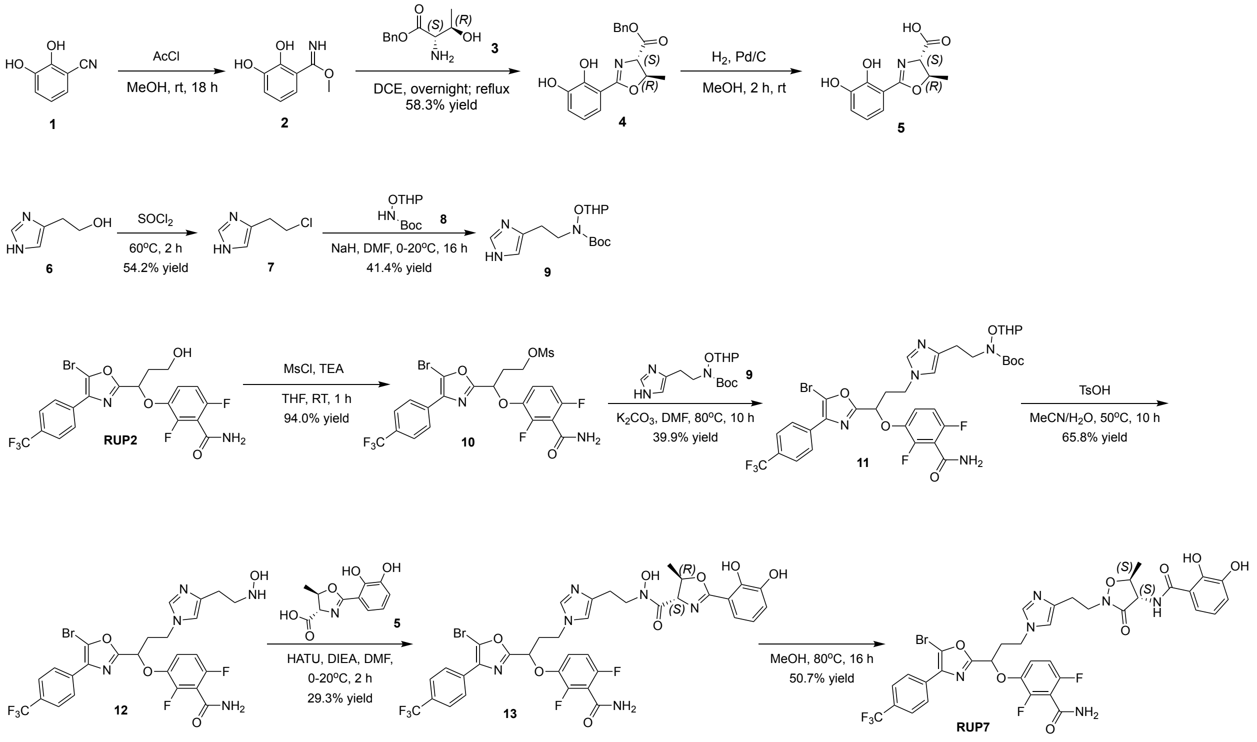

Scheme S1. Synthesis of RUP7.

Methyl 2,3-dihydroxybenzimidate (**2**). To a solution of 2,3-dihydroxybenzonitrile (0.5 g, 3.7 mmol) in MeOH (7 mL) was added acetyl chloride (9.3 g, 118.4 mmol) at 0 °C. The mixture was stirred at room temperature for 16 h. After the reaction was completed, the mixture was quenched with water (20 mL) and adjusted to pH 9 with saturated aqueous $NaHCO_3$. The solution was extracted with EtOAc (20 mL x 3), and the organic layer was dried over sodium sulfate and concentrated under vacuum to give the crude product (0.6 g), which was used for the next step without further purification. LCMS calculated for $C_8H_{10}NO_3$ $(M+H)^+$ m/z = 168.06; found 168.10.

Benzyl(4S,5R)-2-(2,3-dihydroxyphenyl)-5-methyl-4,5-dihydrooxazole-4-carboxylate (**4**). To a solution of 2 (0.7 g, 4.2 mmol) in DCE (10 mL) was added benzyl L-threoninate (0.9 g, 4.2 mmol). The mixture was stirred at 85 °C for 16 h. After the reaction was completed, the mixture was concentrated under vacuum to the crude product, which was purified by flash chromatography (solvent gradient: 0-30% EtOAc in petroleum ether) to afford the desired product (0.8 g, 58.3% yield) as a colorless liquid. LCMS calculated for $C_{18}H_{18}NO_5$ $(M+H)^+$ m/z = 328.11; found 328.0.

(4S,5R)-2-(2,3-dihydroxyphenyl)-5-methyl-4,5-dihydrooxazole-4-carboxylic acid (**5**). To a solution of **4** (0.1 g, 0.31 mmol) in MeOH (5 mL) was added Pd/C (10 mg). The mixture was stirred at room temperature for 2 h under H_2 (30 PSI) atmosphere. After the reaction, the mixture was filtered through celite, and the filtrate was concentrated under vacuum. The crude product (60 mg) was used for the next step without further purification. LCMS calculated for $C_{11}H_{12}NO_5$ $(M+H)^+$ m/z = 238.06; found 238.0.

4-(2-chloroethyl)-1H-imidazole (**7**). A suspension of **6** (2.50 g, 0.022 mol) in $SOCl_2$ (25 mL) was heated to 60 °C for 2 h. TLC (DCM:MeOH = 20:1, R_f = 0.3) showed that the starting material was consumed. The mixture was concentrated under reduced pressure and the crude product was used for the next step without further purification. The product (1.60 g, 12.1 mmol, 54.2% yield) was obtained as a red solid. LCMS calculated for $C_5H_8ClN_2$ $(M+H)^+$ m/z = 131.04; found 131.3.

Tert-butyl(2-(1H-imidazol-4-yl)ethyl)((tetrahydro-2H-pyran-2-yl)oxy)carbamate (**9**). To a solution of **8** (1.60 g, 7.35 mmol) in DMF (16 mL) was added NaH (60% in mineral oil, 306 mg, 7.65 mmol) slowly at 0 °C and stirred for 30 min. **7** (800 mg, 6.12 mmol) in DMF (1 mL) was then added, and the mixture was stirred at 20 °C for 16 h. TLC (DCM:MeOH = 10:1, R_f = 0.5) showed that the starting material was consumed. The mixture was poured into water and then extracted with EtOAc (300 mL x 3). The organic phase was concentrated under reduced

pressure and the crude product was purified by column chromatography (SiO_2, MeOH/DCM = 0-5%) to obtain **9** (800 mg, 2.53 mmol, 41.4% yield). LCMS calculated for $C_{15}H_{26}N_3O_4$ $(M+H)^+$ m/z = 312.19; found 312.2. ^{1}H NMR (400 MHz, DMSO-d_6) δ 11.84 (s, 1H), 7.52 (s, 1H), 6.79 (s, 1H), 4.87 (br m, 1H), 3.97 – 3.85 (m, 1H), 3.80 – 3.66 (m, 1H), 3.62 – 3.47 (m, 5.4Hz, 2H), 2.85 – 2.68 (m, 2H), 1.78 – 1.48 (m, 6H), 1.38 (s, 9H).

3-(5-bromo-4-(4-(trifluoromethyl)phenyl)oxazol-2-yl)-3-(3-carbamoyl-2,4-difluorophenoxy)propyl methanesulfonate (**10**). To a solution of RUP2 (28.3 mg, 0.05 mmol, 1 eq) in anhydrous THF (4 mL) was added TEA (15 µL, 0.11 mmol, 2 eq), followed by MsCl (5 µL, 0.07 mmol, 1.2 eq). The reaction was stirred at room temperature for 1 h. Upon completion, the reaction was diluted with water (30 mL) and extracted twice with DCM (100 mL). The organic layers were then combined and dried over Na_2SO_4 to give **10** as an oil, which was used in the next step without purification. ^{1}H NMR (500 MHz, Chloroform-d) δ 8.05 (dp, J = 8.3, 0.9 Hz, 4H), 7.73 – 7.66 (m, 4H), 7.21 (td, J = 9.1, 5.2 Hz, 2H), 6.87 (td, J = 9.1, 2.0 Hz, 2H), 6.14 (d, J = 18.9 Hz, 4H), 5.39 (dd, J = 9.2, 4.3 Hz, 2H), 4.64 (ddd, J = 10.4, 9.2, 4.0 Hz, 2H), 4.49 (dt, J = 10.2, 4.9 Hz, 2H), 3.79 – 3.63 (m, 5H), 3.12 (qd, J = 7.2, 4.6 Hz, 1H), 3.06 – 2.97 (m, 6H), 2.83 (d, J = 4.0 Hz, 0H), 2.77 – 2.66 (m, 2H), 2.61 – 2.45 (m, 2H), 2.29 – 2.22 (m, 0H), 2.03 (d, J = 9.2 Hz, 0H), 1.45 – 1.36 (m, 3H), 1.29 – 1.20 (m, 2H), 0.89 – 0.82 (m, 1H).

Tert-butyl(2-(1-(3-(5-bromo-4-(4-(trifluoromethyl)phenyl)oxazol-2-yl)-3-(3-carbamoyl-2,4-difluorophenoxy)propyl)-1H-imidazol-4-yl)ethyl)((tetrahydro-2H-pyran-2-yl)oxy)carbamate (**11**). To a solution of **10** (580 mg, 0.966 mmol), **9** (316 mg, 1.01mmol), and K_2CO_3 (200 mg, 1.44 mmol) in DMF (10 mL) was added KI (160 mg, 0.966 mmol). The mixture was then stirred at 80 °C for 10 h. TLC showed that starting material was consumed. The mixture was poured into water, extracted with EtOAc (10 mL x 3), and concentrated under vacuum to give a residue. The crude product was purified by column chromatography (SiO_2, MeOH/DCM = 0-10%) to obtain **11** and the 1,5-substituted imidazole positional isomer in a 2:1 ratio (350 mg, 0.385 mmol,

39.9% yield) as a yellow solid. LCMS calculated for $C_{35}H_{38}BrF_5N_5O_7$ $(M+H)^+$ m/z = 814.18; found 814.05. The compound was used as is for the next step.

3-(1-(5-bromo-4-(4-(trifluoromethyl)phenyl)oxazol-2-yl)-3-(4-(2-(hydroxyamino)ethyl)-1H-imidazol-1-yl)propoxy)-2,6-difluorobenzamide (**12**). To a solution of **11** (350 mg, 0.428 mmol) in MeOH:H_2O (7 mL, v/v = 10:1) was added TsOH (163 mg, 0.857 mmol), and the mixture was stirred at 50 °C for 10 h. LCMS showed that the starting material was consumed completely. The reaction mixture was purified by *prep*-HPLC (Gemini 5 μm C18 150*21.2 mm, MeCN:H_2O (0.1% TFA), Gradient 5-95%) to yield **12** and the 1,5-substituted imidazole positional isomer in a 2:1 ratio (2.10 g, 7.70 mmol, 65.8% yield) as a yellow solid. LCMS calculated for $C_{25}H_{22}BrF_5N_5O_4$ $(M+H)^+$ m/z = 630.08; found 629.95. ^{1}H NMR (400 MHz, DMSO-d_6) δ 10.8 (br s, 1H), 8.87–9.09 (m, 1H), 8.07 (d, J = 8.0 Hz, 2H), 7.89 (d, J = 8.0 Hz, 3H), 7.59 – 7.54 (m, 1H), 7.35 – 7.29 (m, 1H), 7.11 (t, J = 8.4 Hz, 1H), 5.79 – 5.68 (m, 1H), 4.42 (m, 2H), 3.35 (m, 3H), 3.02 – 2.95 (m, 3H), 2.84 – 2.59 (m, 3H).

(4S,5R)-N-(2-(1-(3-(5-bromo-4-(4-(trifluoromethyl)phenyl)oxazol-2-yl)-3-(3-carbamoyl-2,4-difluorophenoxy)propyl)-1H-imidazol-4-yl)ethyl)-2-(2,3-dihyroxyphenyl)-N-hydroxy-5-methyl-4,5-dihydrooxazole-4-carboxamide (**13**). To a solution of **12** (200 mg. 0.316 mmol), **5** (75.15 mg, 0.316 mmol), and DIEA (204 mg, 1.58 mmol) in DMF (4 mL) was added HATU (132 mg, 0.348 mmol) at 0 °C. The mixture was then stirred at 20 °C for 2 h. LCMS showed that **5** was consumed. The reaction mixture was purified by *prep*-HPLC (Gemini 5 μm C18 150*21.2 mm, MeCN:H_2O (0.1% $NH_3{\bullet}H_2O$), Gradient 5-95%) to yield **13** and the 1,5-substituted imidazole positional isomer in a 2:1 ratio (80 mg, 29.3% yield) as a white solid. LCMS calculated for $C_{36}H_{31}BrF_5N_6O_8$ $(M+H)^+$ m/z = 849.13; found 849.1. ^{1}H NMR (400 MHz, DMSO-d_6) d 9.11-9.02 (m, 1H), 8.19-8.01 (m, 3H), 7.88 (d, J = 7.2 Hz, 3H), 7.70-7.45 (m, 1H), 7.28 (d, J = 6.4 Hz, 1H), 7.18-6.87 (m, 2H), 6.77-6.60 (m, 1H), 5.82-5.68 (m, 1H), 5.00 (d, J =

5.6 Hz, 1H), 4.84 (s, 1H), 4.71-4.59 (m, 0H), 4.58-4.33 (m, 3H), 3.08-2.91 (m, 2H), 2.74-2.61 (m, 2H), 1.42-1.36 (m, 3H).

N-((4S,5S)-2-(2-(1-(3-(5-bromo-4-(trifluoromethyl)phenyl)oxazol-2-yl)-3-(3-carbamoyl-2,4-difluorophenoxy)propyl)-1H-imidazol-4-yl)ethyl)-5-methyl-3-oxoisooxazolidin-4-yl(-2,3-dihydroxybenzamide (RUP7). A suspension of **13** (80 mg, 0.0941 mmol) in MeOH (8 mL) was stirred at 80 °C for 16 h. LCMS analysis showed that the starting material was consumed completely. The crude product was purified by *prep*-HPLC (Gemini 5 µm C18 150*21.2 mm, MeCN:H_2O (0.1% TFA), Gradient 5-95%) to yield RUP7 and the 1,5-substituted imidazole positional isomer in a 2:1 ratio (50 mg, 0.15 mmol, 50.7% yield) as a white solid. The product was confirmed by LCMS, calculated for $C_{36}H_{31}BrF_5N_6O_8$ $(M+H)^+$ m/z = 849.13; found 848.95. The mixture was further purified by *prep*-HPLC (Gemini 5 µm NX-C18 150*30 mm, MeCN (0.1% TFA):H_2O (0.1% TFA), Gradient 5-60% in 35 min). ^1H NMR (400 MHz, DMSO-d_6) δ 12.07 (s, 1H), 9.34 (s, 1H), 9.09 (d, *J* = 7.97 Hz, 1H), 8.11 (s, 1H), 8.06 (d, *J* = 8.07 Hz, 2H), 7.91–7.86 (m, 4H), 7.73–7.59 (m, 1H), 7.35–7.28 (m, 1H), 7.26 (d, *J* = 8.07 Hz, 1H), 7.13–7.06 (m, 1H), 6.94 (d, *J* = 7.89 Hz, 1H), 6.74–6.68 (m, 1H), 5.74–5.61 (m, 1H), 4.70 –4.65 (m, 1H), 4.50–4.39 (m, 2H), 3.84–3.70 (m, 2H), 2.96–2.83 (m, 2H), 2.81–2.67 (m, 2H), 2.60–2.58 (m, 1H), 1.37 (d, *J* = 6.10 Hz, 3H). ^{13}C NMR (126 MHz, DMSO-d_6) δ 170.1, 170.1, 168.4, 167.9, 164.2, 162.3, 162.3, 162.3, 161.5, 154.5, 154.5, 152.6, 152.6, 151.1, 150.5, 150.4, 148.5, 148.4, 147.1, 147.1, 141.5, 141.5, 141.4, 141.4, 138.4, 137.9, 137.5, 137.4, 135.6, 135.6, 133.6, 129.5, 129.3, 129.3, 129.1, 129.0, 128.8, 128.0, 127.8, 127.1, 126.9, 126.3, 126.3, 126.3, 126.3, 126.2, 125.6, 125.6, 123.5, 121.3, 120.9, 120.9, 120.9, 119., 119.20, 118.6, 118.2, 118.2, 117.5, 117.3, 117.3, 117.1, 116.5, 116.5, 115.2, 115.2, 115.1, 111.9, 111.7, 78.5, 78.5, 78.2, 73.2, 73.1, 57.5, 57.4, 45.5, 44.5, 42.5, 34.3, 26.0, 21.5, 21.3, 17.1, 17.0. ^{19}F NMR (471 MHz, DMSO-d_6) δ -61.2, -121.3 (dq, *J* = 7.9, 4.2, 3.2 Hz), -132.2 (t, *J* = 9.4 Hz). HRMS, calculated for $C_{36}H_{31}BrF_5N_6O_8$ $(M+H)^+$ m/z = 849.13014; found 849.1307; error: 0.6595 ppm.
