# Supplementary material for: A FtsZ inhibitor-acinetobactin conjugate with enhanced cellular uptake in Acinetobacter baumannii acts synergistically in combination with PBP3-targeting antibiotics
Source: PLoS One. 2025 Oct 14;20(10):e0334409. doi: 10.1371/journal.pone.0334409 (PMC12520410; doi:10.1371/journal.pone.0334409)
Supplement: S1 Fig — (PDF) [file pone.0334409.s002.pdf]

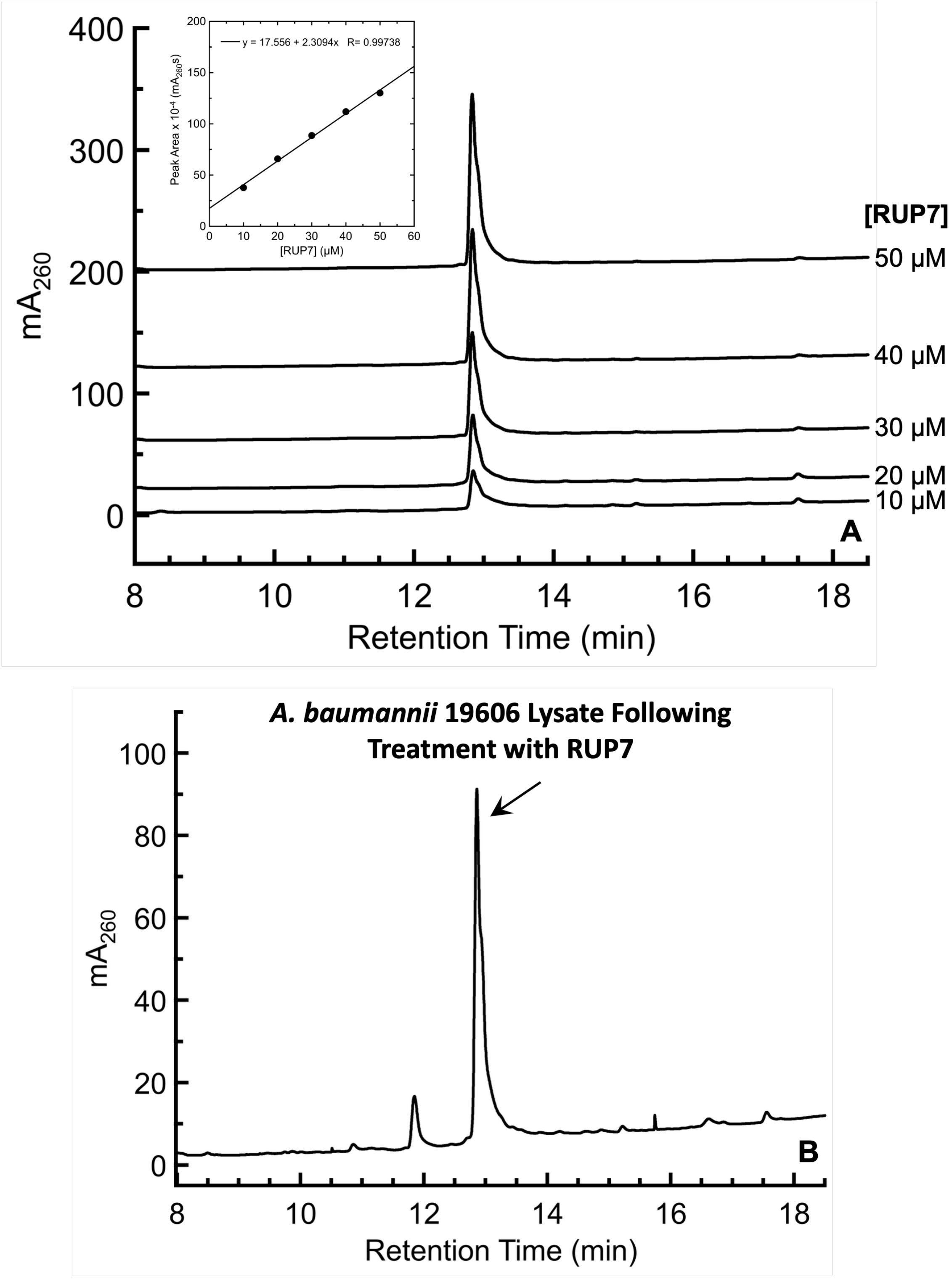

Fig S1. (A) HPLC chromatograms of RUP7 at concentrations ranging from 10 to 50 µM, with the inset showing the corresponding standard curve of peak area as a function of RUP7 concentration. (B) HPLC chromatogram of a representative lysate (from a total of 5 replicate lysates) of *A. baumannii* 19606 cells following treatment with RUP7. The arrow indicates the RUP7 peak.
