# Supplementary material for: A FtsZ inhibitor-acinetobactin conjugate with enhanced cellular uptake in Acinetobacter baumannii acts synergistically in combination with PBP3-targeting antibiotics
Source: PLoS One. 2025 Oct 14;20(10):e0334409. doi: 10.1371/journal.pone.0334409 (PMC12520410; doi:10.1371/journal.pone.0334409)
Supplement: S3 Fig — (PDF) [file pone.0334409.s004.pdf]

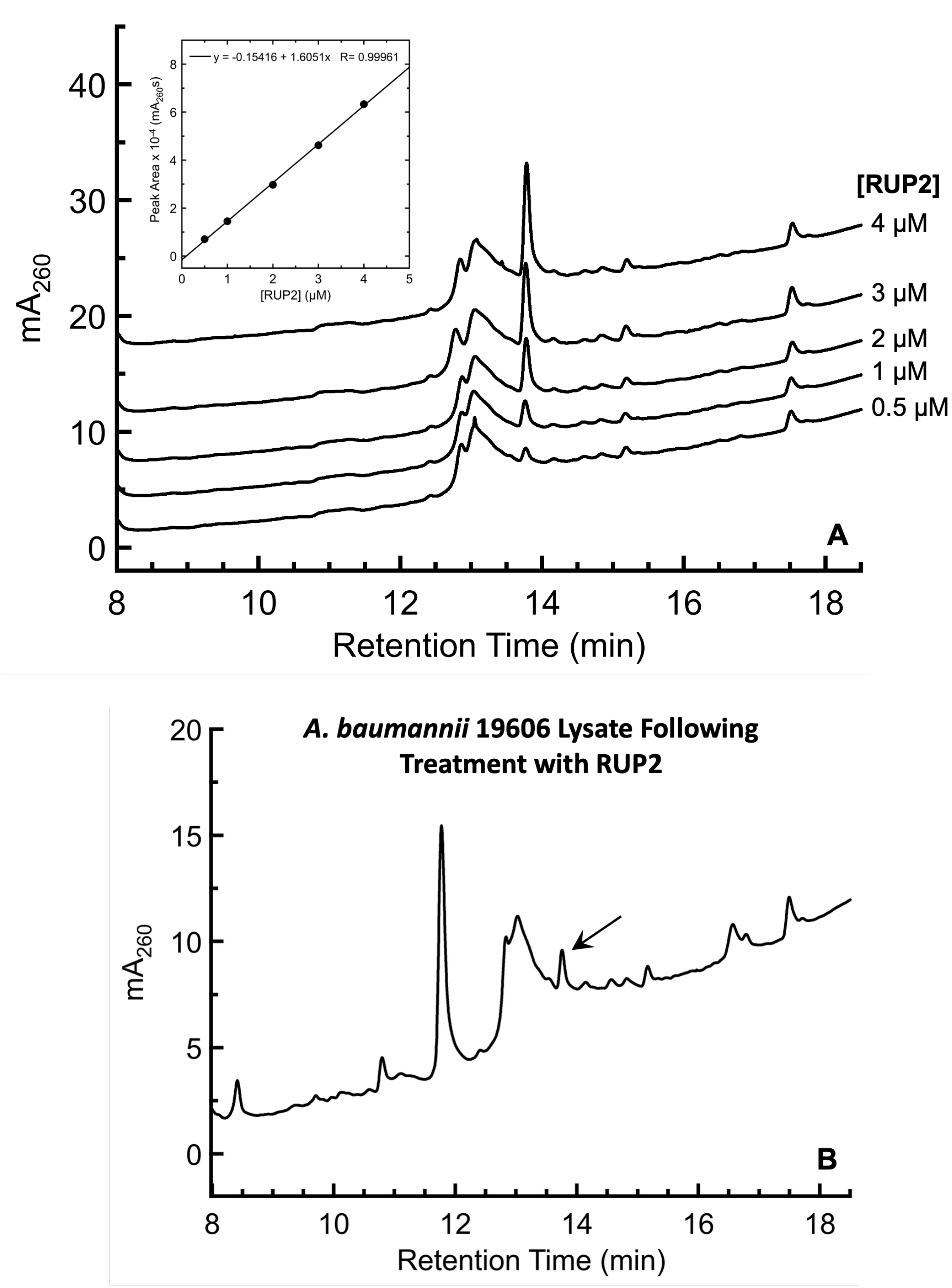

Fig S3. (A) HPLC chromatograms of RUP2 at concentrations ranging from 0.5 to 4 μM, with the inset showing the corresponding standard curve of peak area as a function of RUP2 concentration. (B) HPLC chromatogram of a representative lysate (from a total of 5 replicate lysates) of *A. baumannii* 19606 cells following treatment with RUP2. The arrow indicates the RUP2 peak.
