# Supplementary material for: A FtsZ inhibitor-acinetobactin conjugate with enhanced cellular uptake in Acinetobacter baumannii acts synergistically in combination with PBP3-targeting antibiotics
Source: PLoS One. 2025 Oct 14;20(10):e0334409. doi: 10.1371/journal.pone.0334409 (PMC12520410; doi:10.1371/journal.pone.0334409)
Supplement: S4 Fig — (PDF) [file pone.0334409.s005.pdf]

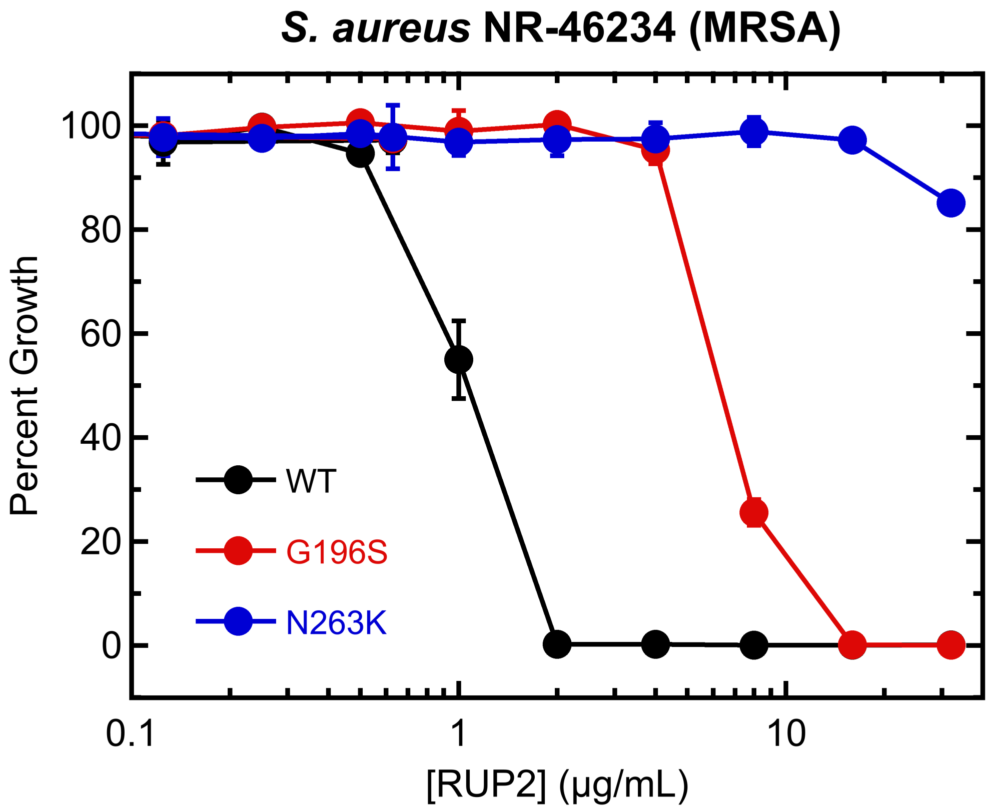


**Fig S4.** Antibacterial activity of RUP2 against wild-type (WT) *S. aureus* NR-46234 (MRSA) and mutant strains expressing either G196S or N263K mutant FtsZ. All strains were assayed in CAMH media. Each experimental datapoint represents the average of three replicates, with the error bars reflecting the standard deviations from the mean.
