# Supplementary material for: A FtsZ inhibitor-acinetobactin conjugate with enhanced cellular uptake in Acinetobacter baumannii acts synergistically in combination with PBP3-targeting antibiotics
Source: PLoS One. 2025 Oct 14;20(10):e0334409. doi: 10.1371/journal.pone.0334409 (PMC12520410; doi:10.1371/journal.pone.0334409)
Supplement: S5 Fig — (PDF) [file pone.0334409.s006.pdf]

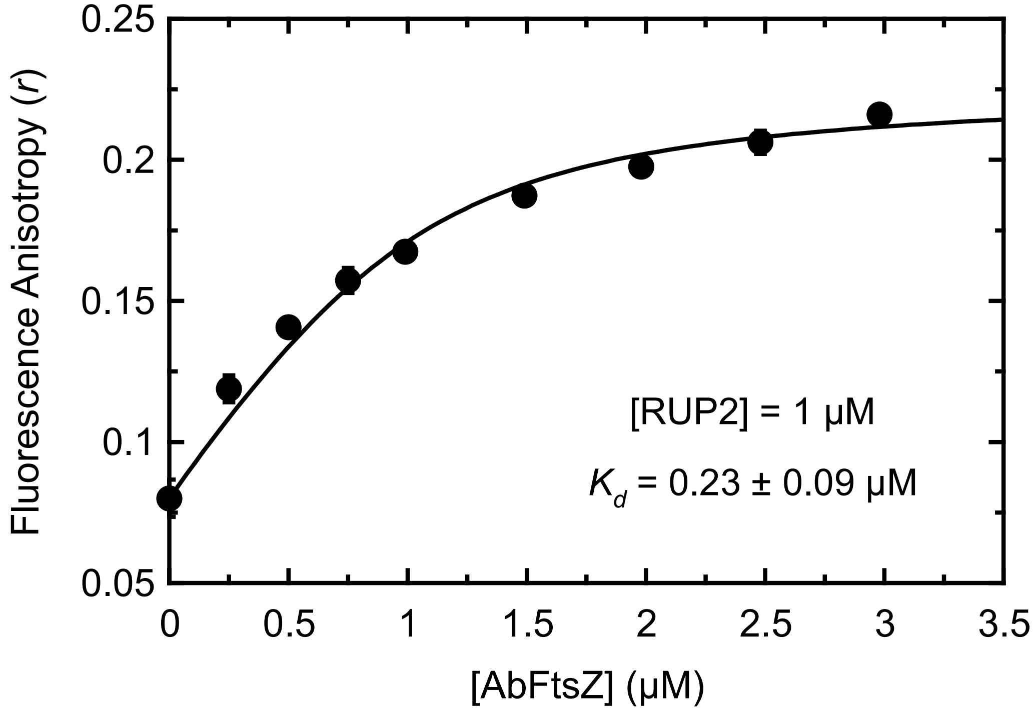

Fig S5. Fluorescence anisotropy (*r*) of 1 μM RUP2 as a function of increasing concentrations of AbFtsZ. Each experimental datapoint represents the average of five replicates, with the error bars reflecting the standard deviations from the mean. The solid line represents a non-linear least squares fit of the *r* values using Equation (3), with the indicated K_d value being derived from this fit.
