# Supplementary material for: A FtsZ inhibitor-acinetobactin conjugate with enhanced cellular uptake in Acinetobacter baumannii acts synergistically in combination with PBP3-targeting antibiotics
Source: PLoS One. 2025 Oct 14;20(10):e0334409. doi: 10.1371/journal.pone.0334409 (PMC12520410; doi:10.1371/journal.pone.0334409)
Supplement: S1 Table — (PDF) [file pone.0334409.s007.pdf]

Table S1. Sequences of the oligonucleotide primers used in the qPCR studies of *A. baumannii* 19606 cells grown in CAMH media, M9 media, or M9 media + 10 μM Fe^{3+}.

Name	Sequence (5′ → 3′)
AbBauA_qPCR_F	ATCATGTTGATGGCGTGACACG
AbBauA_qPCR_R	GGTTTCAACAGAGCCCCAATCC
AbBauB_qPCR_F	GACCGAACTGCTGTGATGGAAC
AbBauB_qPCR_R	CGCATCGGCATCGACAAAAATG
AbBauC_qPCR_F	TGCGTTGCTTTTTCCTCGATTG
AbBauC_qPCR_R	CTGGCTTAATCCATGCGTTCCC
AbBauD_qPCR_F	GCTCGGGGCTGAACAAATACAG
AbBauD_qPCR_R	TACGGCTGGCTGTAAGAGTGAG
AbBauE_qPCR_F	GAGCAAGATCGTCAGGTGGTTG
AbBauE_qPCR_R	AAGTACGTCTGTTTGCTGTGCG
AbBasB_qPCR_F	AATGTGCCATTGGTGCTGACAG
AbBasB_qPCR_R	GGCTCATTTCTGGTTCAGCGAG
AbBasD_qPCR_F	CACCCGCCAAATATGGAAGTGG
AbBasD_qPCR_R	GAACAGGTGAGAGCTGAATCGC
AbBarA_qPCR_F	ATGATGCACAACTTGAGCAGGC
AbBarA_qPCR_R	AGCTGAGGATAAAAACGCACGC
AbBarB_qPCR_F	CAAGCTGGGTTCTCATCAGTGC
AbBarB_qPCR_R	TGGCACGAGCTAAAGATTTGCC
AbRpoB_qPCR_F	AGCTTCTTTTGTTGGTGGCTCG
AbRpoB_qPCR_R	ACATTACGTGCTGACTTGACGC
AbRpoD_qPCR_F	GTACGGCAGCAGCTTCTTCTTC
AbRpoD_qPCR_R	GTATTCCTGTACACGAGCGTGC
